# Evidence for unconscious priming using a Bayesian single-subject approach

**DOI:** 10.1093/nc/niag052

**Published:** 2026-08-20

**Authors:** Nicolás Sánchez-Fuenzalida, Simon van Gaal, Zazie van den Hurk, Timo Stein, Johannes J Fahrenfort

**Affiliations:** Department of Applied and Experimental Psychology, Free University Amsterdam, Van der Boechorststraat 7, 1081 BT Amsterdam, The Netherlands; Institute for Brain and Behavior Amsterdam, Free University Amsterdam, Van der Boechorststraat 7, 1081 BT Amsterdam, The Netherlands; Department of Applied and Experimental Psychology, Free University Amsterdam, Van der Boechorststraat 7, 1081 BT Amsterdam, The Netherlands; Institute for Brain and Behavior Amsterdam, Free University Amsterdam, Van der Boechorststraat 7, 1081 BT Amsterdam, The Netherlands; Department of Psychology, University of Amsterdam, Nieuwe Achtergracht 129-B, 1018 WS Amsterdam, The Netherlands; Amsterdam Brain & Cognition, University of Amsterdam, Nieuwe Achtergracht 129-B, 1018 WS Amsterdam, The Netherlands; Department of Applied and Experimental Psychology, Free University Amsterdam, Van der Boechorststraat 7, 1081 BT Amsterdam, The Netherlands; Institute for Brain and Behavior Amsterdam, Free University Amsterdam, Van der Boechorststraat 7, 1081 BT Amsterdam, The Netherlands; Department of Psychology, University of Amsterdam, Nieuwe Achtergracht 129-B, 1018 WS Amsterdam, The Netherlands; Amsterdam Brain & Cognition, University of Amsterdam, Nieuwe Achtergracht 129-B, 1018 WS Amsterdam, The Netherlands; Department of Applied and Experimental Psychology, Free University Amsterdam, Van der Boechorststraat 7, 1081 BT Amsterdam, The Netherlands; Institute for Brain and Behavior Amsterdam, Free University Amsterdam, Van der Boechorststraat 7, 1081 BT Amsterdam, The Netherlands; Department of Psychology, University of Amsterdam, Nieuwe Achtergracht 129-B, 1018 WS Amsterdam, The Netherlands; Amsterdam Brain & Cognition, University of Amsterdam, Nieuwe Achtergracht 129-B, 1018 WS Amsterdam, The Netherlands

**Keywords:** unconscious processing, awareness, consciousness, single-subject, Bayesian statistics, unconscious priming

## Abstract

Many studies have claimed the existence of unconscious priming, but a valid statistical procedure for substantiating such claims is often lacking. Absence of prime awareness is often inferred from non-significant p-values, and priming and awareness are typically tested by comparing two separate tests. In a classic study by Vorberg, D., Mattler, U., Heinecke, A., Schmidt, T., & Schwarzbach, J. (Different time courses for visual perception and action priming. Proceedings of the National Academy of Sciences 2003, 100(10), 6275–6280.), observers were slower to identify the direction of an arrow mask when it followed an arrow prime pointing in the opposite direction, despite being unable to identify the prime direction. Using this study as a starting point, we addressed several methodological shortcomings that are common in the field. First, we used a Bayesian methodological-statistical framework to test priming effects and prime awareness at the single-subject level across multiple sessions. Second, participants received extensive training in the prime awareness task to ensure they understood and executed the task as intended. Third, we directly compared prime awareness and priming. Using this approach, we found an indirect-task advantage consistent with unconscious priming at short prime-mask stimulus onset asynchronies (SOAs) in three out of six participants, under the assumption that the awareness task was at least as sensitive as the priming task. However, unlike in the original study by Vorberg and colleagues, evidence for unconscious priming was not consistently present across all SOAs, and awareness increased with SOA. Priming effects were highly consistent across participants, whereas awareness showed substantial inter-individual variability.

Significance Statement By combining a Bayesian framework with extensive training and direct comparison of priming and awareness, this study provides robust evidence for an indirect-task advantage, consistent with unconscious priming under the assumption that the awareness task was at least as sensitive as the priming task. Unlike Vorberg, D., Mattler, U., Heinecke, A., Schmidt, T., & Schwarzbach, J. (Different time courses for visual perception and action priming. Proceedings of the National Academy of Sciences 2003, 100(10), 6275–6280.) original study, unconscious priming was not consistently present across stimulus onset asynchronies (SOAs), as prime awareness increased with prime-mask SOA. Further, robust priming effects coexisted with highly variable awareness across individuals, emphasizing the need to evaluate unconscious processing at the individual level rather than relying solely on group averages. This study advances the field by showing how unconscious priming can be tested with a single-subject approach and a direct comparison of awareness and priming.

## Introduction

**Figure 1:**
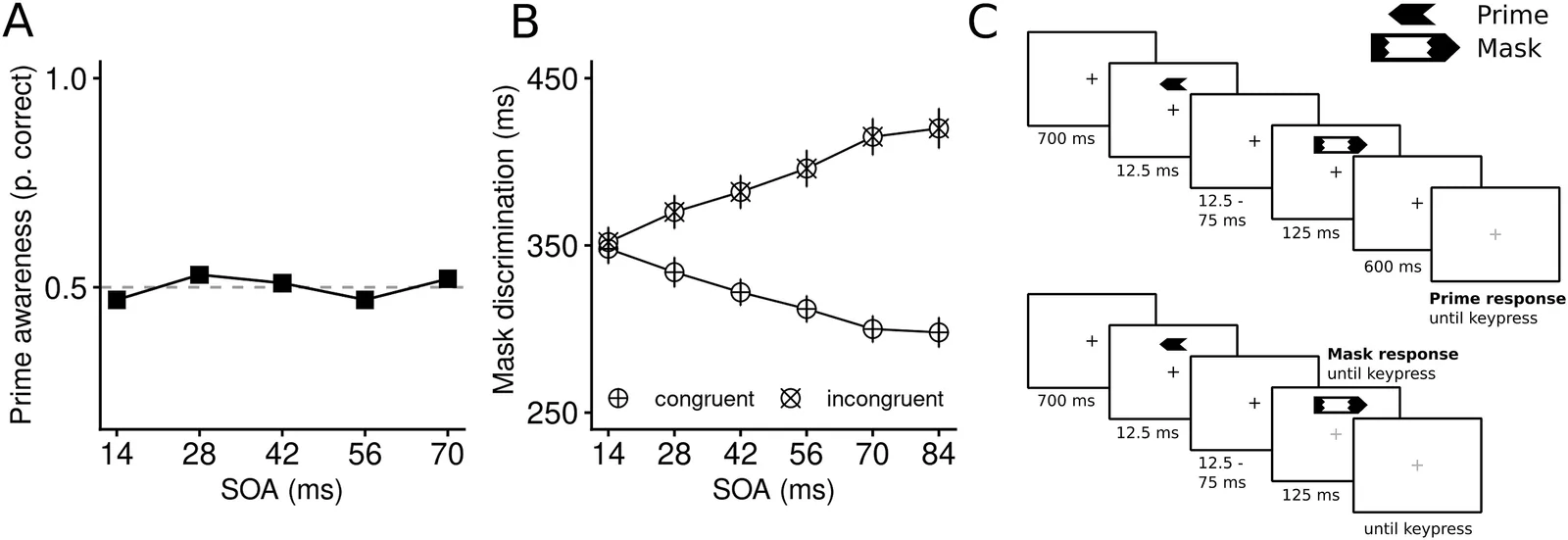
Data pattern in the original results by Vorberg et al. (2003) and study paradigm. (A) Qualitative data pattern of the prime discrimination results in Vorberg et al. (2003). Group-level proportion of correct responses for each SOA. The dotted line at *P* = .5 indicates chance performance. For the original data see Fig. 2C in Vorberg et al. (2003). (B) Qualitative data pattern of the mask discrimination results in Vorberg et al. (2003). Group-level average RT (ms) on correct trials for each SOA, separately for congruent and incongruent trials. For the original data see Fig. 1B in Vorberg et al. (2003). Note that plots (A) and (B) do not depict empirical data, but approximate estimations that mimic the qualitative pattern of results as shown in Figs. 2C and 1B in Vorberg et al. (2003), reproduced here only for illustration purposes. (C) the experimental paradigm used by Vorberg et al. (2003) with SOA timings as used in the current experiment. For the original SOA timing see Fig. 1A in Vorberg et al. (2003). All trials started with a fixation period of 700 ms, followed by the prime (12.5 ms), followed by a variable SOA period (12.5–75 ms or 300 ms), followed by the mask (125 ms). In prime discrimination trials (top), the mask was followed by a 600 ms fixation period after which the fixation cross turned light grey to signal that participants could respond. In mask discrimination trials (bottom), the fixation turned light grey as soon as the mask was presented to signal that participants could respond. The fixation remained on screen until a response was registered.

The existence of priming without awareness is a central claim in unconscious cognition research, with reports dating back more than half a century (Eriksen 1960). Unconscious priming occurs when the identity of an invisible prime stimulus affects the response to a subsequent stimulus (indirect effect), despite the prime not being consciously perceived (absence of a direct effect) (Schmidt and Vorberg 2006). For example, in a classic study by Vorberg et al. (2003), observers were faster to identify the direction of an arrow-shaped mask when it followed an arrow-shaped prime pointing in the same (rather than the opposite) direction (priming effect), despite them not being able to discriminate the direction of the prime (prime awareness, see Fig. 1A and B). Although many studies have claimed such unconscious priming effects, the topic remains controversial, as claims of unconscious priming often rely on statistical tests that fail to adequately demonstrate the absence of awareness.

Most studies supporting the existence of unconscious priming rely on what is usually referred to as a dissociation procedure (Reingold and Merikle 1988, Hannula et al. 2005, Schmidt and Vorberg 2006), i.e. showing that prime awareness is not significantly different from chance, while also showing a significant priming effect. One issue with this approach is establishing true absence of awareness. Typically, researchers ask participants to detect or discriminate a masked prime across many trials and then calculate an aggregated measure of performance, such as proportion correct or Signal Detection Theory’s d’. The performance is often quite low and not statistically distinguishable from chance (i.e. *P* > .05), which is then interpreted as evidence for absence of awareness. Although this is common practice, non-significant p-values are not sufficient evidence to claim the absence of an effect, as they may also reflect insufficient power to detect a small non-zero effect (Keysers et al. 2020). Fortunately, this problem can be addressed by using either alternative frequentist (Lakens 2017) or Bayesian (Rouder et al. 2009, Dienes 2014) methods that can provide evidence for the absence of an effect. Note also that some studies may remove participants performing above chance to then test priming on a post-hoc selected subsample. This practice is problematic because selecting subjects based on the same data used to demonstrate the absence of awareness is comparable to “double-dipping” (Kriegeskorte et al. 2009) and likely to confound the results with regression to the mean (Shanks 2017, Stein et al. 2024).

Further, many unconscious priming studies, including Vorberg et al. (2003), rely on what is known as the double *t-*test approach. In this approach, the comparison of two experimental effects is performed by executing two separate *t*-tests and independently establishing significance for each. When one turns out to be significant and the other is not, one concludes that they are different (Nieuwenhuis et al. 2011). This practice is also common in unconscious priming studies, where awareness and priming are tested independently and the results of these tests are then combined to conclude the presence of unconscious priming. However, this approach is fundamentally flawed (Nieuwenhuis et al. 2011, Meyen et al. 2022). Instead, in a proper statistical framework, priming and prime awareness should be compared directly on the same scale and within the same statistical test (Meyen et al. 2022, Stein et al. 2024).

Another often overlooked issue is that awareness is frequently assessed by asking participants to complete a difficult task (e.g. discriminating briefly presented masked stimuli) with little or no practice. In this context, it is not straightforward to judge whether a participant is performing at chance due to the difficulty of the task or because they do not know how to perform the task, even though the relevant information to perform the task is still consciously perceived. Additionally, even if participants are trained and know in principle how to perform the task, some of them may still give up on awareness trials if the task is so difficult that they feel they cannot perform the task meaningfully (i.e. performing above chance). Awareness could be present in both cases but not reflected in their prime discrimination responses.

To address the aforementioned issues, we attempted to replicate the study by Vorberg et al. (2003) using a Bayesian framework to test for unconscious priming at the single subject level across multiple experimental sessions. First, participants completed a control session that was as similar as possible to Experiment 1 in Vorberg et al. (2003) (tasks, stimulus presentation, stimulus design, and practice; see Methods for minor differences) before completing our version of the experiment. This session was merely intended to see whether we could replicate the pattern of results that Vorberg et al. (2003) obtained. Next, we collected data in a number of ensuing sessions, in which we modified aspects of the design to address specific issues: (i) Participants were thoroughly instructed and practised each task before completing the experiment, in contrast to Vorberg et al. (2003), where participants received minimal instructions and did not practice the tasks. (ii) Instead of performing one task per session, as in Vorberg et al. (2003), we alternated prime discrimination blocks and mask discrimination blocks in the same session (see Fig. 1C) to control for session-level confounds, such as differences in motivation or fatigue. (iii) To make trial-feedback constant across tasks, auditory feedback was given in the mask discrimination task and in the prime discrimination task, unlike in Vorberg et al. (2003), where mask discrimination feedback was visual and prime discrimination feedback was auditory. (iv) As in Vorberg et al. (2003), we collected the data of six participants across multiple sessions. However, instead of performing a frequentist fixed effect analysis at the group level as Vorberg et al. (2003) did, we tested at the single subject level using a Bayesian statistical framework. This approach allowed us to explicitly test for the absence of awareness separately for each participant. Further, instead of testing a fixed number of sessions, each participant completed consecutive sessions until our Bayesian statistical framework indicated that there was sufficient evidence for or against unconscious priming based on a pre-defined stopping rule.

With these changes in place we determined whether unconscious priming can be demonstrated at the single subject level using a statistically and methodologically principled method.

## Methods

### Experiment 1

**Ethics**. All experimental procedures were approved by the Ethical Review Board (VCWE) of the Free University Amsterdam (ref. number VCWE-2021-173).

**Participants and sample size.** As in Vorberg et al. (2003), six participants (one man and five women, 19.8 years old on average, SD = 1.33) were recruited through the Free University Amsterdam lab pool and were rewarded money or research credits for each 90-min (first session) or 60-min (session two onwards) session. No participants were excluded from analysis.

**Optional stopping.** Analyses were conducted at the single-subject level using a Bayesian sequential testing framework and a pre-defined stopping rule. After session one, each participant completed consecutive experimental sessions until there was strong evidence for either an effect (BF_10_  *\ge* 10) or no effect (BF_10_  *łe* 0.1) in at least four out of six prime-mask stimulus onset asynchronies (SOAs) (see *Stimuli and timing*) both in the prime discrimination and in the mask discrimination task (see *Analysis* for a detailed description of the prime and mask analyses). Thus, further data collection sessions were added for each participant until these criteria would be met. In this Bayesian testing framework optional stopping is not considered problematic (Rouder 2014). Only sessions two and onwards were included in the analysis relevant for the application of the optional stopping rule. The decision to collect data until four out of six prime-mask SOAs met the stopping rule was made in order to keep data collection manageable, given that larger samples are often needed to obtain the same strength of evidence for the null than for the alternative hypothesis (Stefan et al. 2019). Moreover, based on (Vorberg et al. 2003) we expected most SOAs in the prime discrimination task to show null or near-zero effects. Pilot data suggested that four out of six SOAs were sufficient to establish whether a participant performed at chance at any of the SOAs, and whether priming was present at those same SOAs.

**Stimuli, timing and apparatus.** Prime and mask stimuli consisted of left- or right-pointing arrows. The prime subtended ${1.86}^{{}^{\circ}}\times{0.8}^{{}^{\circ}}$ of visual angle and the mask subtended ${3.47}^{{}^{\circ}}\times{1.09}^{{}^{\circ}}$. Stimuli were presented over a white background centred horizontally, 1.38° above or below the fixation. The prime was fully black whereas the mask was black with an inner white cutout. The contour of the prime was adjacent to the contour of the inner cutout of the mask. Based on the visual material and specifications in the methods of Vorberg et al. (2003), we reconstructed the stimuli to be as close as possible to the original. On each trial the prime was presented for 12.5 ms and the mask for 125 ms, with a variable SOA of 12.5, 25, 37.5, 50, 62.5 or 75 ms. Due to the refresh rate of the monitors available, the duration of the prime, mask and SOAs were different but within the range of the original study (Experiment 1 in Vorberg et al. 2003). From session two onwards, we added a condition with a long SOA of 300 ms to reduce the overall difficulty of the experiment and to provide some trials in which the relevant feature of the stimulus was clearly visible (see *General Procedure* for the full rationale). Stimuli were presented on a 240 Hz LCD monitor (Asus ROG Strix XG258Q 1080p) with a resolution of 1920x1080 pixels. The width of the monitor was 52.6 cm, and participants viewed the display from a distance of 80 cm using a chin rest.

**Trial layout and Tasks.** Each trial started with a 700 ms fixation, followed by the prime (12.5 ms), a variable SOA (12.5 to 75 in steps of 12.5 ms, or 300 ms), and then the mask (125 ms). In the first session, we tried to replicate the core original experiment by Vorberg et al. (2003) as closely as possible (albeit with slightly different presentation times and different display technology; see *Stimuli, timing and apparatus* above). Participants performed a combined mask discrimination and prime detection task, and a prime discrimination task. In the combined mask discrimination and prime detection trials, the fixation cross turned light grey at the mask onset and participants were instructed to indicate the direction of the mask as quickly as possible by pressing one of two keys on the keyboard (A for left or L for right). 250 ms after their response, participants were prompted to indicate whether the prime was presented by pressing one of two keys on the keyboard (A for absent or L for present; see Supplementary Fig. S1). Primes were presented in two thirds of the trials. Visual feedback was given after incorrect mask discrimination responses. In prime discrimination trials, the fixation cross turned light grey 600 ms after the offset of the mask to prompt participants to answer as accurately as possible the direction of the prime by pressing one of two keys on the keyboard (A for left or L for right; see Fig. 1C). Auditory feedback (100 ms) was given after incorrect prime discrimination responses. This setup was identical to sessions 2 and 3 (mask discrimination and prime detection) and 6 and 7 (prime discrimination) of Experiment 1 in Vorberg et al. (2003), except for the precise timing of the stimuli (see Stimuli and timing of Experiment 1) and the use of an LCD instead of a CRT monitor (see Discussion). From session two onwards, participants discriminated the direction of the prime (as in session one) and the direction of the mask (as in session one, but primes were present in all trials and no prime detection response was required). Auditory feedback (100 ms) was given in both tasks immediately after incorrect responses. In all sessions, mask discrimination trials were aborted and repeated within the same block if no response was registered within 700 ms from the mask offset.

**General procedure.** In the first session, participants were instructed to indicate the direction of the mask (mask discrimination task) as quickly as possible, and to indicate as accurately as possible whether the prime was presented (prime detection task). After the instructions, participants completed 12 blocks of 72 trials of the combined mask discrimination and prime detection task. At the end of each block, participants were informed about the percentage of correct responses in that block (separately for mask discrimination and prime detection responses) and the average reaction time (RT) (only for mask discrimination responses). An extra message prompting participants to answer more accurately was displayed if their mask discrimination performance dropped below 90%. Similarly, a message prompting participants to answer faster was displayed if their average mask discrimination RT dropped below 400 ms. In the second part of the session, participants were instructed to indicate the direction of the prime (prime discrimination task) as accurately as possible. Then, they completed 12 blocks of 72 trials of the prime discrimination task. At the end of each block participants were informed about the percentage of correct responses in that block. Participants were allowed to take a short break at the end of each block if they wished to do so, and they had two mandatory 3-min breaks (after one and two thirds of the session). The position of the stimuli (above or below the fixation), prime presence, the direction of the prime and mask, as well as all SOA conditions were fully counterbalanced at the block level.

In session two, participants received more detailed instructions than in session one and completed an extensive practice block for each task before starting the experiment. To ensure participants were executing the prime discrimination as intended, we devised a progressively difficult practice block in which participants could practice experiment-like trials (12.5–75 ms SOA) while also having to perform perfectly on easy trials (300 ms SOA). First, participants were required to give 10 correct responses in a row on easy trials. Then, they completed 10 easy trials and 20 difficult trials and were required to answer all easy trials correctly. Finally, they completed 30 difficult trials without any performance requirement. In the practice of the mask discrimination task, participants were required to give 10 correct responses in a row in trials identical to the experimental trials. All participants were able to successfully complete the practice requirements. From session three onwards, participants received instructions and completed a 30-trial warm-up block for each task before starting the experiment. The warm-up blocks were identical to the experimental blocks. Given that Vorberg et al. (2003) reported chance performance across all SOAs in the prime discrimination task, we also considered the possibility that participants knew how to perform the task but disengaged because they perceived the task to be too difficult. To reduce the chances of participants giving up, we included a set of trials with a long SOA (300 ms), in which the relevant feature of the prime stimulus participants was clearly visible.

After the instructions, and practice or warm-up, participants completed six blocks of 112 trials for each task (prime or mask discrimination) in alternating order. The experimental blocks were identical across sessions. Half of the participants started with a mask discrimination block while the other half started with a prime discrimination block. The first block of the remaining sessions alternated between prime and mask discrimination blocks depending on the first block of the previous session of the same participant. At the beginning of each block, participants were instructed whether they had to indicate the direction of the prime or the direction of the mask. At the end of each block, participants received block-level feedback identical to the feedback in session one. Participants were allowed to take a short break at the end of each block if they wished to do so.

In total, participants completed 1728 trials in the first session, and 1344 trials in each remaining session. Without considering session one, participants one and six completed six sessions (4032 trials per task), participants two, three, four and five completed five sessions (3 360 trials per task). The position of the stimuli (above or below fixation), the direction of the prime and mask, as well as all SOA conditions were fully counterbalanced at the block level.

**Analysis.** To test prime discrimination at the single subject level, we fitted a logistic regression model to the prime discrimination trial-level data separately for each participant and each SOA. In the model, the reported direction of the prime (*response*: left/right) was predicted by the direction of the prime stimulus (*stimulus*: left/right) and session.


$$\mathrm{response}\sim \mathrm{stimulus}+\mathrm{session}$$

For the intercept, the regression coefficient of *stimulus* (prime direction), and the regression coefficient of *session*, we used independent normal distributions centred on 0 with a standard deviation of 5 (Normal(0, 5)), which are weakly informative.

To test prime discrimination at the group level, we fitted the same model but including a random intercept for each *participant*. For the prior of the participant-specific intercepts, we use a Student’s *t* distribution with 3 degrees of freedom, a location of 0, and a scale of 2.5 as prior (Student-t(3, 0, 2.5)), constrained to be non-negative.


$$\mathrm{response}\sim \mathrm{stimulus}+\mathrm{session}+\left(1\mid \mathrm{participant}\right)$$

For the analysis of the first session, *session* was not included in the model.


$$\mathrm{response}\sim \mathrm{stimulus}+\left(1\mid \mathrm{participant}\right)$$

It should be noted that while random slopes are desirable in larger samples, doing so in our group-level models led to estimation and convergence issues, which is not surprising considering the small number of participants in the current low-N, intensive-measurement design. Instead, we use the single-subject level models to account for individual differences.

To test the priming effect at the single subject level, we fitted a regression model to the mask discrimination trial-level data separately for each participant and each SOA. In the model, RT was predicted by the congruency between prime and mask (*congruency*: congruent/incongruent) and session.


$$\mathrm{rt}\sim \mathrm{congruency}+\mathrm{session}$$

For the intercept, the regression coefficient of *congruency* (congruent/incongruent), and the regression coefficient of *session*, we used independent normal distributions centred on 0 with a standard deviation of 5 (Normal(0, 5)), which are weakly informative.

To test the priming effect at the group level, we fitted the same model but including a random intercept for each *participant*. For the prior of the participant-specific intercepts, we use a Student’s *t* distribution with 3 degrees of freedom, a location of 0, and a scale of 2.5 as prior (Student-t(3, 0, 2.5)), constrained to be non-negative


$$\mathrm{rt}\sim \mathrm{congruency}+\mathrm{session}+\left(1\mid \mathrm{participant}\right)$$

For the analysis of the first session, *session* was not included in the model.


$$\mathrm{rt}\sim \mathrm{congruency}+\left(1\mid \mathrm{participant}\right)$$

To directly test prime discrimination and priming, we fitted a logistic regression model to the prime discrimination and RT-recoded mask discrimination trial-level data separately for each participant and each SOA. The model specifications were identical to the model used to test for prime discrimination, except that *accuracy* (correct/incorrect) was predicted by *task* (prime discrimination or mask discrimination) and session.


$$\mathrm{accuracy}\sim \mathrm{task}+\mathrm{session}$$

Priors were identical to the priors used in the prime discrimination logistic regression. For the group-level analysis, we followed the same logic as in the group-level analysis of the prime discrimination task.


$$\mathrm{accuracy}\sim \mathrm{task}+\mathrm{session}+\left(1\mid \mathrm{participant}\right)$$

Finally, to assess heterogeneity across participants in prime discrimination and priming, we fitted a logistic regression model to the prime discrimination and RT-recoded mask discrimination data from all participants and SOAs, except for the 300 ms SOA. *Accuracy* (correct/incorrect) was predicted by the interaction between *soa_c* (mean-centred SOA) and *task* (prime discrimination or mask discrimination), and *session*. The model also included a random intercept for each *participant* and a random slope of *soa_c* for each *participant* × *task* combination.


\begin{align*}\mathrm{accuracy}\sim&\, \,\mathrm{soa}\_\mathrm{c}\ast \mathrm{task}+\mathrm{session}+\left(1\mid \mathrm{participant}\right)\\&+\left(0+\mathrm{soa}\_\mathrm{c}\mid \mathrm{participant}:\mathrm{task}\right) \end{align*}

Note that the variance across participants was estimated separately for each task as:


\begin{align*} \left(0+\mathrm{soa}\_\mathrm{c}\mid \mathrm{participant}:\mathrm{task}\right) =&\,\left(0+\mathrm{soa}\_{\mathrm{c}}_{\mathrm{prime}}\mid \mathrm{participant}\right)\\ &+\left(0+\mathrm{soa}\_{\mathrm{c}}_{\mathrm{mask}}\mid \mathrm{participant}\right) \end{align*}

For the prior of the regression coefficient of *soa_c* (mean-centred SOA), *task*, and *session*, we used independent normal distributions centred on 0 with a standard deviation of 5 (Normal(0, 5)), which are weakly informative. For the prior of the participant-specific intercepts and *soa_c* slopes, we use a Student’s *t* distribution with 3 degrees of freedom, a location of 0, and a scale of 2.5 as prior (Student-t(3, 0, 2.5)), constrained to be non-negative. Note that for this analysis we mean-centred the predictor SOA to improve model fit.

**Model fitting.** To fit the models we used the *brms* R package (version 2.23.0) for Bayesian Multilevel Models (Bürkner 2017). For each model we ran four Markov chain Monte Carlo (MCMC) chains with between 5000 and 10000 iterations, depending on model complexity. All models had a thinning factor of 1 and warm-up iterations were always half of the total iterations. Model convergence was assessed using the $\hat{R}$ convergence diagnostic ($\hat{R}$ < 1.01) and by visually inspecting trace plots using the ‘hairy caterpillar’ criterion (Roy 2020).

**Bayes Factors.** To derive Bayes Factors from the prime discrimination and priming analyses we used the Savage-Dickey density ratio method, which compares the density of the model-sampled prior and posterior distributions at zero (no-effect). In the prime discrimination task, the posterior distribution was sampled using the *stimulus* coefficient (prime direction), whereas the *congruent* coefficient (prime and mask congruency) was used in the mask discrimination task. This effectively estimates whether the effect of the direction of the prime or the mask-prime congruency is zero. For the direct comparison the posterior distribution of the *task* coefficient was sampled, effectively estimating whether there was a difference between the prime and mask discrimination task. When the posterior distribution did not overlap with zero, the Savage-Dickey ratio yielded an infinite Bayes Factor. We therefore report these cases as BF_10_ > 10^40^. Bayes Factors are labelled according to the guidelines proposed by Lee and Wagenmakers (2013): anecdotal evidence (1 < BF_10_ < 3), moderate evidence (3 ≤ BF_10_ < 10), strong evidence (10 ≤ BF_10_ < 30), very strong evidence (30 ≤ BF_10_ < 100) and extreme evidence (100 ≤ BF_10_). To obtain the equivalent thresholds for evidence for the null (BF_01_) the reciprocal value can be used; for instance, anecdotal evidence for the null corresponds to 0.33 < BF_10_ < 1.

## Results

In different blocks, participants indicated the direction of the prime (prime discrimination task) or the direction of the mask (mask discrimination task) by pressing one of two keys on a keyboard (see Fig. 1C). Prime and mask discrimination tasks differed only with respect to which stimulus was the target. From these two tasks, we derived, separately for each prime-mask SOA, a measure of prime awareness (proportion of correct responses on the prime discrimination task) and a priming measure (difference in RT in the mask discrimination task between congruent and incongruent trials). Following Vorberg et al. (2003), in all mask discrimination analyses only correct trials were included and the slowest and fastest RTs were removed (independently for each participant and congruency condition). Note that the percentage of removed trials was not reported in the original manuscript, thus we opted for a conservative trimming (1% slowest and 1% fastest; we performed a robustness check by trimming RTs using two extra different thresholds and showed that the results are robust against different trimming thresholds; see Supplementary Text S1 and Supplementary Tables S1, S2, S3, S4, and  S5 for the results).

**Figure 2:**
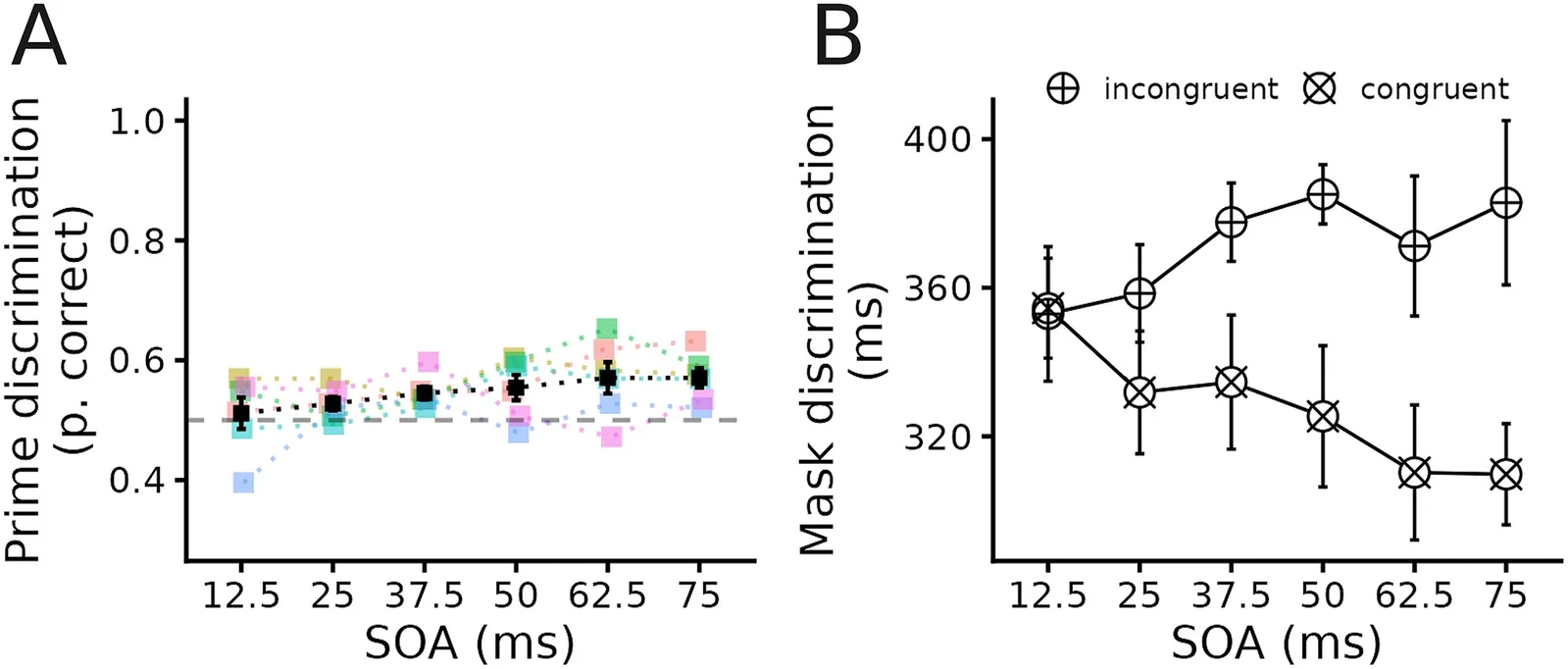
Control experiment results. (A) Prime discrimination results. Proportion of correct responses per participant (colour) and group-level (black) average for each SOA. (B) Mask discrimination results. Group-level average rt (ms) on correct trials for each SOA, separately for congruent and incongruent trials. Error bars indicate the SEM.

First, we show the results from the first session, in which participants performed the task in a similar fashion to the original experiment, with little to no training. We did not intend to perform extensive statistical analysis on these data with lower trial counts. These data were collected merely to show that the data pattern from Vorberg et al. (2003) could be recreated, prior to making modifications to the training and design. As can be seen in Fig. 2, the overall data pattern was similar to that obtained in their study (see Fig. 1A and B). When participants had to discriminate the direction of the prime, there was anecdotal to very strong evidence for chance performance at the three shortest SOAs (12.5 ms: BF_10_ = 0.035, *d* = 0.05; 25 ms: BF_10_ = 0.104, *d* = 0.13; 37.5 ms: BF_10_ = 0.772, *d* = 0.2), whereas there was moderate to extreme evidence for above-chance performance at the three longest SOAs (50 ms: BF_10_ = 4.59, *d* = 0.25; 62.5 ms: BF_10_ = 10^5^, *d* = 0.32; 75 ms: BF_10_ = 33, *d* = 0.32; see Fig. 2A). Note, however, that even at the longest SOA, the effect size did not exceed 0.32, suggesting that participants performed barely above chance. When discriminating the direction of the mask, except for the shortest SOA (12.5 ms: BF_10_ = 0.002, *d* = 0.11), participants showed reliable priming effects, i.e. they were faster on congruent compared to incongruent trials (25 ms: BF_10_ = 10^15^, *d* = 0.75; 37.5 ms: BF_10_ > 10^40^, *d* = 1.01; 50 ms: BF_10_ = 10^37^, *d* = 1.08; 62.5 ms: BF_10_ > 10^40^, *d* = 1.27; 75 ms: BF_10_ = 10^17^, *d* = 1.86; see Fig. 2B). Vorberg et al. (2003) also performed a prime detection task which they did not analyse (see their Fig. 2B). In our version of this task the false alarm rate was similar across SOAs (~4%) and the hit-rate went from roughly 50% to 90% across SOAs, suggesting participants could detect the prime across all SOAs (see Supplementary Fig. S2). Next, we wanted to establish whether this data pattern would hold after introducing more extensive instructions and training and after implementing our statistical framework (see *Methods*).

**Figure 3:**
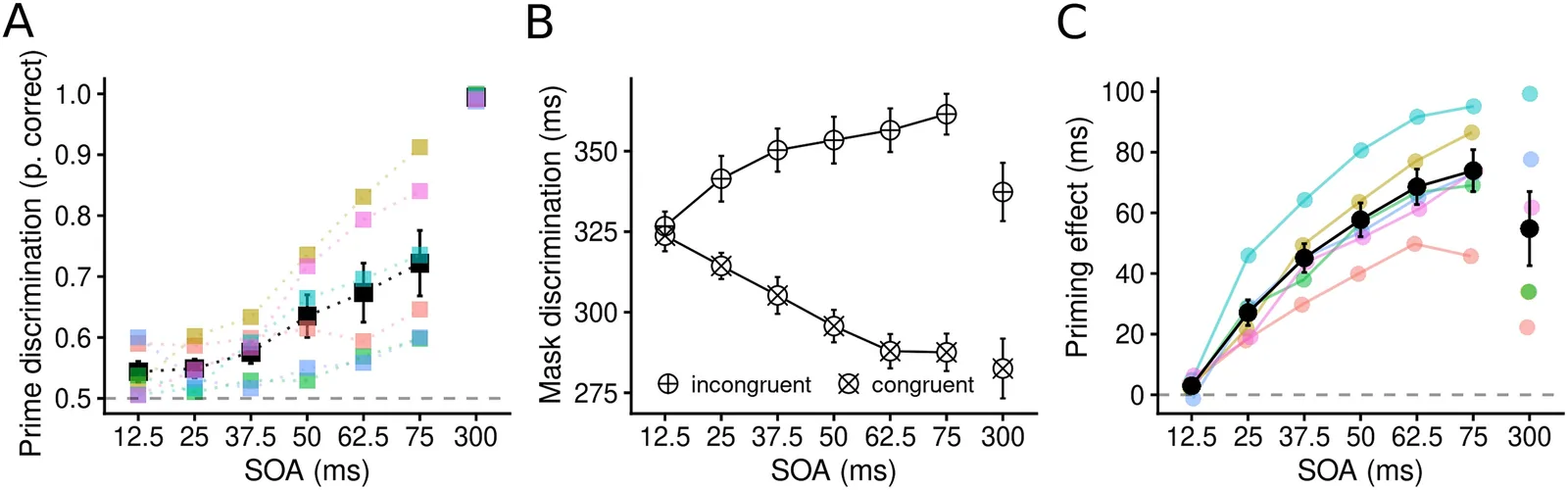
Experiment 1 group-level results. (A) Prime discrimination results. Proportion of correct responses per participant (colour) and group-level (black) average for each SOA. (B) Mask discrimination results. Group-level average RT (ms) on correct trials for each SOA, separately for congruent and incongruent trials. (C) Priming effect. Participant (colour) and group-level (black) average difference in RT (ms) on correct trials between congruent and incongruent trials in the mask discrimination task. Error bars indicate the SEM.

For ease of comparison with the results in Vorberg et al. (2003) and from the initial session, we first show the group results before turning to the single subject data. Figures 3A, 3B and 3C depict the group-level results of the prime and mask discrimination tasks. Unlike Vorberg et al. (2003), at the group level, participants were able to discriminate the direction of the prime above chance across all SOAs, and unlike in the original study this ability also strongly increased with SOA (12.5 ms: BF_10_ = 10^7^, *d* = 0.2; 25 ms: BF_10_ = 10^15^, *d* = 0.2; 37.5 ms: BF_10_ > 10^40^, *d* = 0.3; 50 ms: BF_10_ = 10^14^, *d* = 0.6; 62.5 ms: BF_10_ > 10^40^, *d* = 0.8; 75 ms: BF_10_ = 10^28^, *d* = 1.1; 300 ms: BF_10_ > 10^40^, *d* = 6.6; see Fig. 3A). However, note that, in terms of effect size, participants performed barely above-chance at the two shortest SOAs (12.5 and 25 ms: *d* ≤ 0.3). The mask discrimination results closely resembled the original results by Vorberg et al. (2003): RTs were, on average, longer for incongruent compared with congruent trials (see 3B), and this priming effect difference became more pronounced as the SOA increased (12.5 ms: BF_10_ = 0.009, *d* = 0.1; 25 ms: BF_10_ = 10^19^, *d* = 0.8; 37.5 ms: BF_10_ = 10^13^, *d* = 1.4; 50 ms: BF_10_ = 10^13^, *d* = 1.7; 62.5 ms: BF_10_ = 10^13^, *d* = 2; 75 ms: BF_10_ = 10^13^, *d* = 2; 300 ms: BF_10_ = 10^14^, *d* = 1.3; see Fig. 3C).

**Figure 4:**
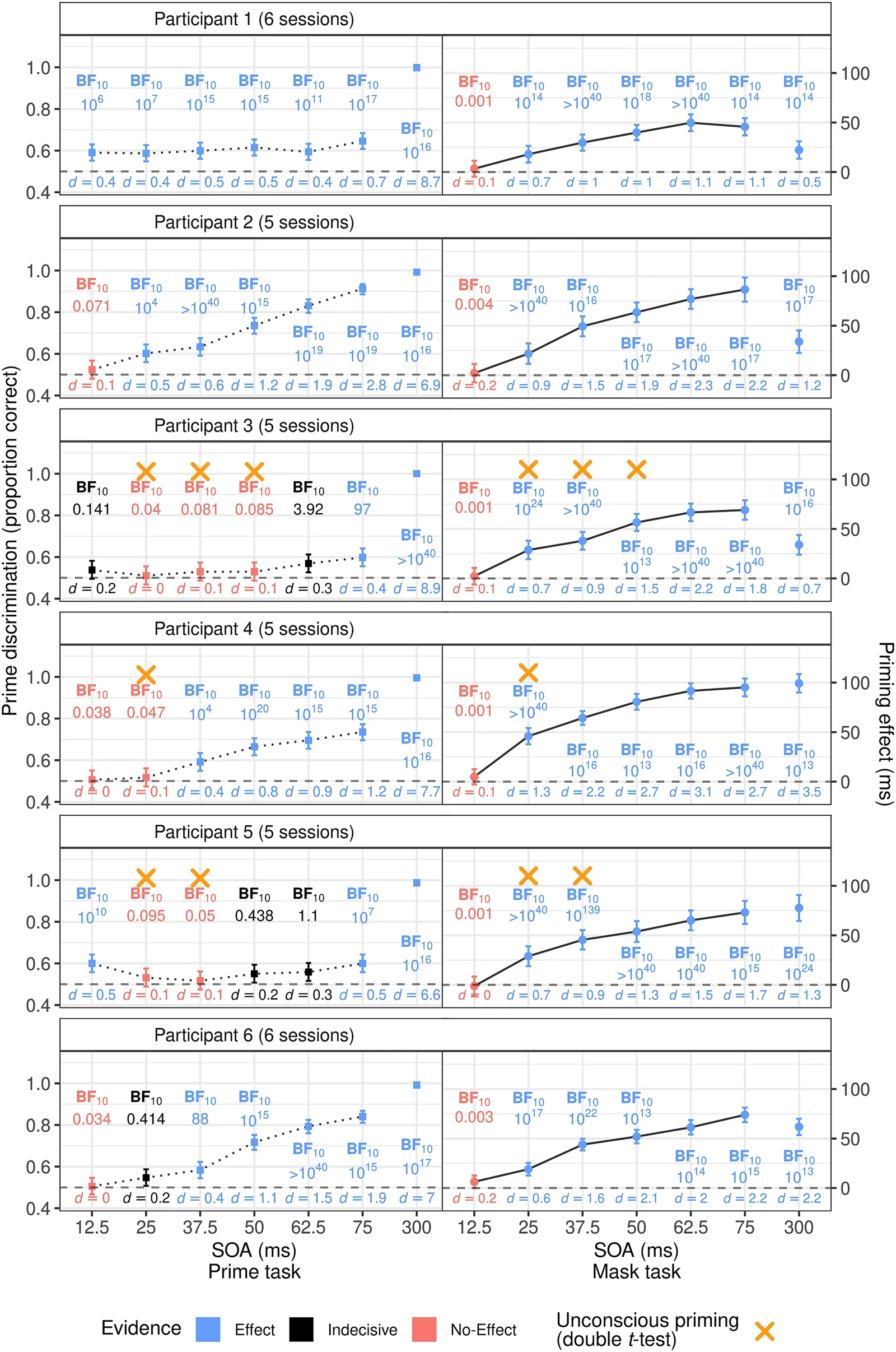
Experiment 1 subject-level results. Each panel depicts the prime (left) and mask (right) discrimination results for each participant and each SOA. For the prime discrimination task, the proportion of correct responses is plotted. For the mask discrimination task, the average correct trial rt (ms) difference between incongruent and congruent trials is plotted. A golden X indicates SOAs for which the participant was at chance in the prime discrimination task and had a priming effect in the mask discrimination task if evaluated using the double *t-*test criterion that Vorberg et al. (2003) adopted. All error bars indicate 95% credible interval.

Next, we investigated these findings at the single subject level. Figure 4 depicts the subject-level data for each SOA in the prime discrimination (left panels) and mask discrimination (right panels) tasks. The data of participant 1 revealed extreme evidence for above-chance performance across all SOAs (Bayes Factor and Cohen’s *d* values can be found in Fig. 4 and in Supplementary Table S6). Participant 2, on the other hand, exhibited strong evidence for chance performance at the shortest SOA (12.5 ms), and extreme evidence for above-chance performance across all other SOAs. Participant 3 showed moderate to strong evidence for chance performance at the four shortest SOA (12.5, 25, 37.5 and 50 ms), and moderate to extreme evidence for above-chance performance at the remaining SOAs. For participant 4, there was strong evidence for chance performance at the two shortest SOAs (12.5 and 25 ms), and extreme evidence for above-chance performance at the remaining SOAs (37.5–300 ms). Participant 5 exhibited extreme evidence for above-chance performance at the shortest SOA (12.5 ms), strong evidence for chance performance at 25 and 37.5 ms, anecdotal evidence for chance performance at 50 ms, anecdotal evidence for above-chance performance at 62.5 ms and extreme evidence for above-chance performance at 75 ms and 300 ms. Participant 6 showed strong evidence for chance performance at 12.5 ms, anecdotal evidence for chance performance at 25 ms, and very strong to extreme evidence for above-chance performance on the remaining SOAs. To summarize, there was considerable variability in how well participants were able to perform the prime discrimination task. Further, most participants performed at chance or near-chance at the two shortest SOAs, with their performance improving at longer SOAs. Very strong evidence for above-chance performance (BF_10_ ≥ 30) was predominant at the 37.5 ms SOA.

In contrast to the prime discrimination results, the priming effect in the mask discrimination task was very consistent across participants. As in the original study by Vorberg et al. (2003), all participants showed extreme evidence for the absence of a priming effect at the shortest SOA (12.5 ms: BF_10_ < 0.01, *d* ≤ 0.2), while there was extreme evidence for a priming effect in all the remaining SOAs (25–75 ms: BF_10_ > = 10^13^, *d* > = 0.6; Bayes Factor and Cohen’s *d* values can be found in Fig. 4 and in Supplementary Table S7). When considering the prime discrimination and priming effect results together, three participants met the original Vorberg-style criterion for unconscious priming in at least one SOA, i.e. evidence for chance-level prime discrimination together with evidence for a priming effect. This corresponds to the double *t-*test logic used in the original study by Vorberg et al. (2003), implemented here as two Bayesian *t*-tests. Participant 3 at the 25, 37.5 and 50 ms SOAs, participant 4 at the 25 ms SOA, and participant 5 at the 25 and 37.5 ms SOAs (see Fig. 4; unconscious priming by the double *t-*test criterion is denoted with a golden X).

We also evaluated whether the inclusion of a 300 ms SOA had an effect on participants’ decision criterion compared to the original version of the experiment, as it has been argued that differences in perceived difficulty and stimuli range influence decision criterion placement (Lockhead 2004, King and Dehaene 2014, Mill et al. 2014). We compared the decision criterion of each participant in sessions 1 and 2, and we found strong to very strong evidence (BF_10_ < 0.06) for no difference in decision criterion (see Supplementary Text S2).

Although we found evidence for unconscious priming at the single subject level for some participants and some SOAs, these findings are based on the problematic double *t-*test approach. The combination of two Bayesian *t*-tests (one to determine the presence or absence of awareness and another to determine the presence of a priming effect) essentially suffers from the same problem as executing two frequentist *t*-tests: the comparison of two experimental effects requires a statistical test on their difference (Nieuwenhuis et al. 2011). The statistically proper way to compare the awareness and priming effects is to directly compare both effects to establish what has been dubbed an ‘indirect task advantage’ (where the prime discrimination task is termed the direct task, while the priming task is termed the indirect task; Meyen et al. 2022). Thus, we set out to test whether there are SOAs for which the priming task has an advantage over the awareness task.

However, doing so requires putting results from the two tasks on the same scale to compare them directly. To accomplish this, and in line with previous work (Schmidt 2002, Meyen et al. 2022), we recoded the RT data of the mask discrimination task as accuracy using the median-split technique, which consists in labelling congruent trials with RTs shorter than the RT median and incongruent trials with RTs longer than the median as correct responses, and the remaining trials as incorrect responses. Note that in our case, we calculated the median RT separately for each session. This transformation allowed us to directly test prime discrimination and priming effect within the same scale and statistical test. In this framework, unconscious processing occurs if there is an indirect task advantage (priming effect) over the direct task (prime discrimination), which would indicate the indirect task is capitalizing on information that is otherwise not available consciously.

**Figure 5:**
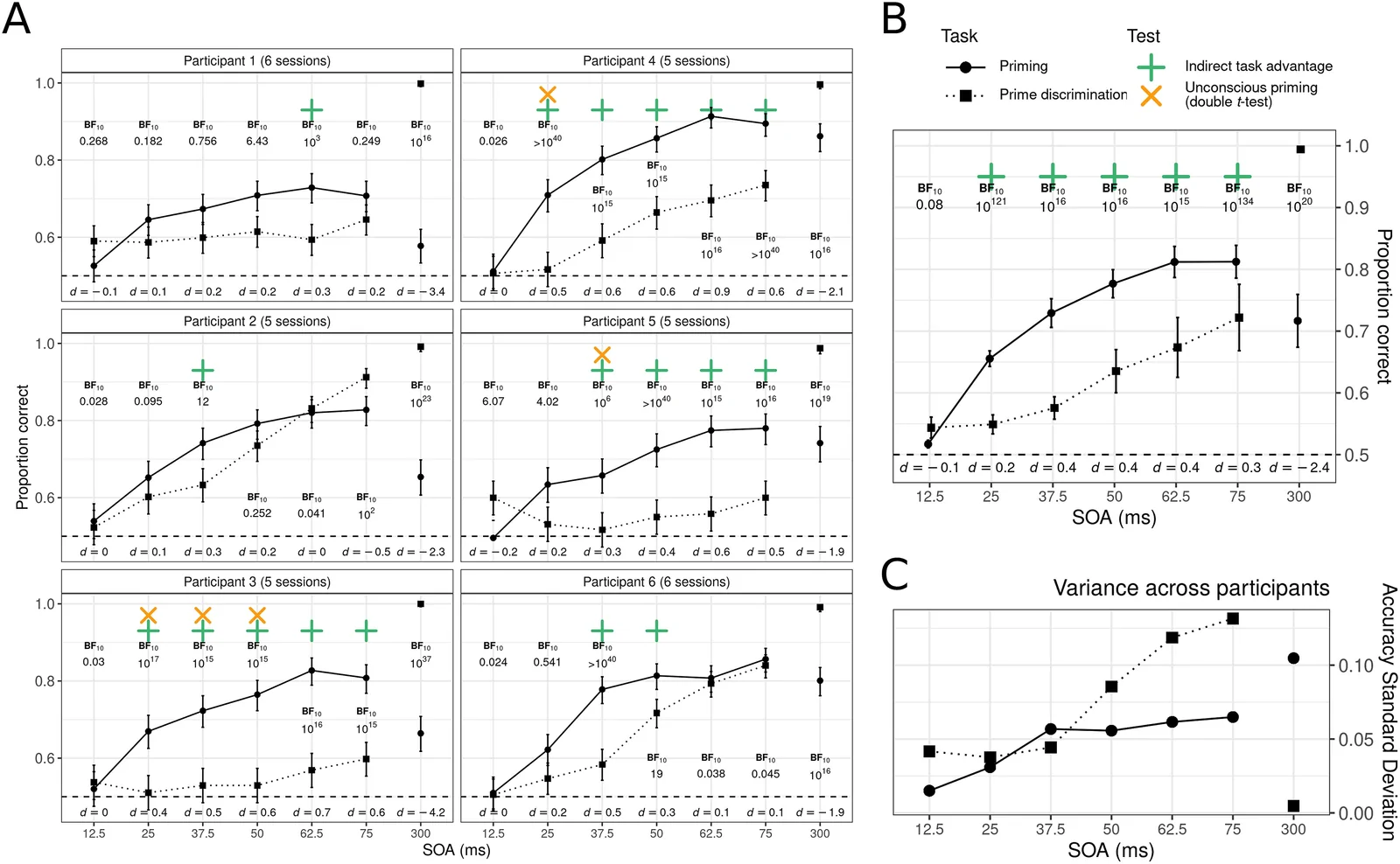
Experiment 1 direct comparison results. (A) Subject- and (B) group-level direct comparison. Proportion of correct responses in the prime discrimination task and priming effect (mask discrimination task) recoded as proportion of correct responses. SOAs with an indirect task advantage (priming > prime discrimination) are marked with a green cross. SOAs showing unconscious priming (double *t-*test approach) are marked with a golden X. While an indirect task advantage is present both at the subject and group level, unconscious priming (double *t-*test approach) is only present at the subject level. Error bars indicate 95% credible interval in subject-level results and SEM in group-level results. C) Variance across participants in prime discrimination and in the priming effect.

Figure 5 depicts the sensitivity of the prime discrimination task and the sensitivity of the priming task. At the single subject-level (Fig. 5A), participant 1 showed anecdotal to moderate evidence for the absence of an indirect task advantage at four SOAs (12.5, 25, 37.5 and 75 ms) and moderate to extreme evidence for an indirect task advantage at 50 and 62.5 ms (Bayes Factor and Cohen’s *d* values can be found in Fig. 5A and in Supplementary Table S8). At the 300 ms SOA there was extreme evidence for a larger effect on prime discrimination compared to priming, and this was true for all participants. For participant 2, there was moderate to very strong evidence for the absence of an indirect task advantage at most SOAs (12.5, 25, 50 and 62.5), strong evidence for an indirect task advantage at the 37.5 ms SOA and extreme evidence for a direct task advantage at the 75 ms SOA. For participant 3 and 4, there was very strong evidence for the absence of an indirect task advantage at the shortest SOA and extreme evidence for an indirect task advantage in all other SOAs. Participant 5 exhibited moderate evidence for a direct task advantage at the shortest SOAs and moderate to extreme evidence for an indirect task advantage at the remaining SOAs (25–75 ms). Finally, participant 6 showed strong to extreme evidence for an indirect task advantage at the 37.5 and 50 ms SOAs and anecdotal to very strong evidence for the absence of an indirect task advantage at the remaining SOAs (12.5, 25, 62.5 and 75 ms). When pooling the data of all subjects, there was strong evidence for no difference between tasks at the shortest SOA (12.5 ms: BF_10_ = 0.08, *d* = −0.1) and extreme evidence for an indirect task advantage at all remaining SOAs (25–75 ms: BF_10_ > 10^10^, *d* > = 0.2; see Fig. 5B). As with the single subject-level result, the 300 ms SOA resulted in extreme evidence for a direct task advantage, i.e. for prime discrimination exceeding the priming effect (BF_10_ = 10^20^, *d* = 2.4). To summarize, both subject- and group-level results offer strong evidence for an indirect task advantage across all subjects and multiple SOAs. Further, the three participants that showed unconscious priming when evaluated using the double *t-*test approach, also showed an indirect task advantage in at least one SOA.

Note that dichotomizing continuous mask discrimination RTs into binary responses using the median-split technique has been criticized for potentially resulting in information loss. To evaluate potential information loss, we estimated the median correlation between raw priming RT and median-split transformed values separately for each participant but across SOAs and sessions (see Supplementary Fig. S3). The correlation was strong across all participants (*r* between 0.84 and 0.93). To further evaluate the convergence of the transformed and raw priming values we fitted a model in which the recoded RT values were predicted by raw priming values, SOA and session across participants (see Supplementary Text S3 for a detailed description of the analysis; see Supplementary Fig. S4). There was extreme evidence (BF_10_ > 10^40^) that raw priming values predicted recoded priming values, after accounting for SOA and session, with an R^2^ of 0.68 (95% CI [0.60 0.75]). Overall, these results suggest a strong convergence between raw and transformed values.

**Figure 6:**
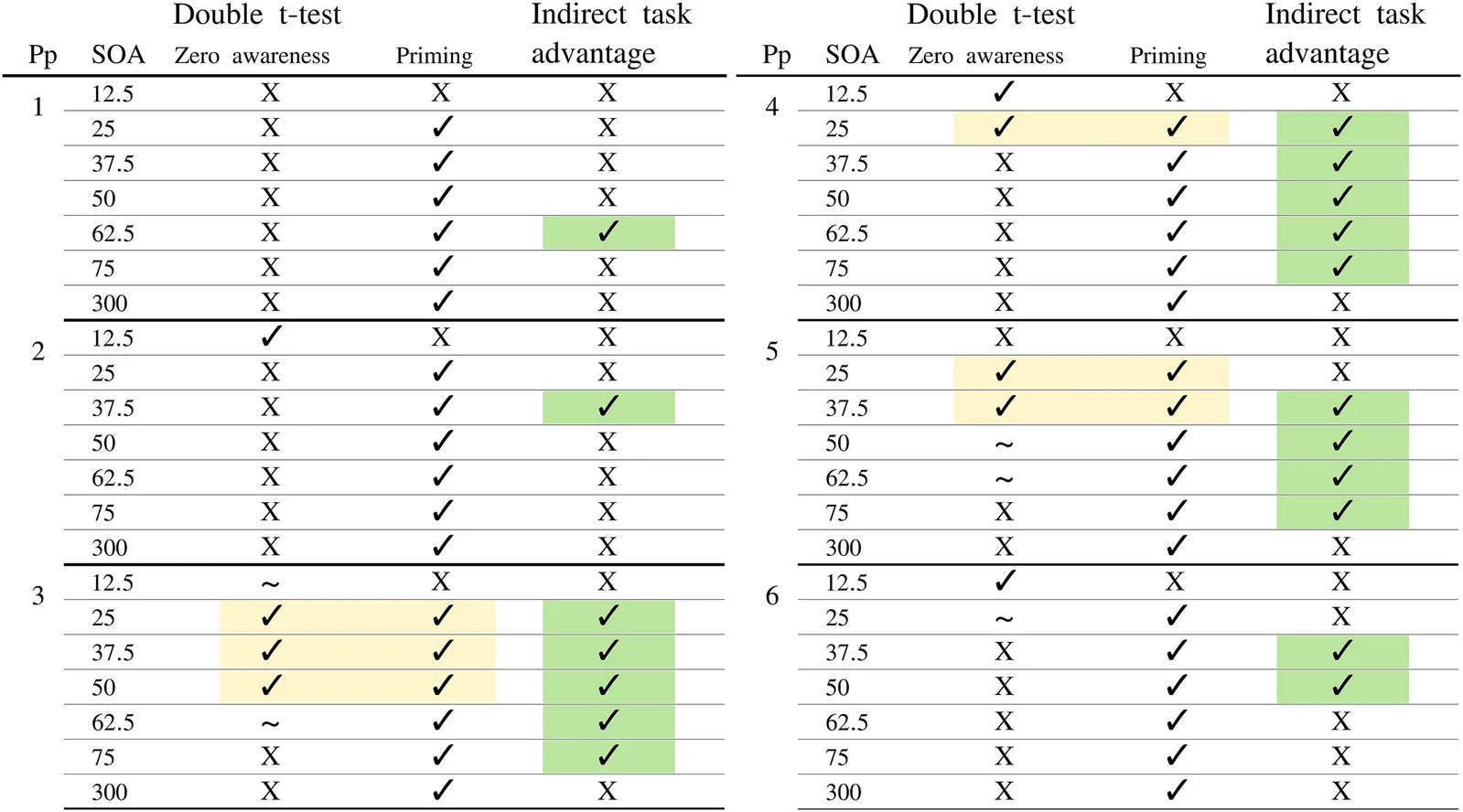
Summary outcomes double *t-*test approach and direct comparison. For each participant and SOA it is indicated whether the zero-awareness and priming criteria are met (double *t-*test) and whether there is an indirect task advantage (priming larger than awareness). Check and X symbols depict strong evidence (BF_10_ > = 10 or BF_10_ < = 0.1), tilde (~) indicates inconclusive evidence according to the evidence threshold we set (0.1 < BF_10_ < 10).

Finally, as mentioned before, performance in the prime discrimination task varied greatly, whereas the priming effect was very consistent across participants. Indeed, when calculating the average standard deviation across subjects for each SOA and task, there was more variance in the prime discrimination task in most SOAs (see Fig. 5C). Only at the longest SOAs there was more variance in the priming effect than in the prime discrimination task, reflecting ceiling performance in the prime discrimination task at the 300 ms SOA. Importantly, the two SOAs that showed the least to no variance difference between the prime and mask discrimination task are also the SOAs that show unconscious priming (double *t-*test) and an indirect task advantage in three participants (see Fig. 6 for a summary of the outcomes of the double *t-*test approach and direct comparison).

To formally test this claim we fitted a model in which we directly quantified and compared the variance between participants across SOAs on each task. There was moderate evidence (BF_10_ = 6) for the variance being larger in the prime discrimination task compared to the mask discrimination (RT-recoded) task (see Supplementary Fig. S5). To rule out that the difference in variance was due to low reliability in one of the tasks we evaluated the internal consistency of each task by performing 5000 random splits and calculated the average Spearman-Brown corrected reliability separately for each SOA and task (see Supplementary Fig. S6). In the prime discrimination task, the Spearman-Brown corrected reliability estimate was at least 0.74 in all SOAs except for the 300 ms SOA, for which it was 0.6 (12.5 ms = 0.79 95% CI [0.3, 0.97]; 25 ms = 0.74 [0.15, 0.96]; 37.5 ms = 0.82 [0.41, 0.97]; 50 ms = 0.96 [0.87, 0.99]; 62.5 ms = 0.98 [0.93, 1]; 75 ms = 0.99 [0.95, 1]; 300 ms = 0.62 [−0.33, 0.96]). In the mask discrimination task (RT-recoded responses), the reliability was the lowest for the two shortest SOAs (12.5 ms = −0.53 95% CI [−0.89, 0.68]; 25 ms = 0.63 [−0.19, 0.95]), whereas for the rest it was above 0.9 (37.5 ms = 0.91 95% CI [0.71, 0.99]; 50 ms = 0.92 [0.72, 0.99]; 62.5 ms = 0.94 [0.8, 0.99]; 75 ms = 0.95 [0.82, 0.99]; 300 ms = 0.97 [0.9, 1]). To summarize, reliability was medium to high for most SOAs. The main exceptions were the 300 ms SOA in the prime discrimination task, where performance was close to ceiling, and the shortest SOA in the RT-recoded mask discrimination task, where we found no priming effect.

## Discussion

Using a stringent single subject Bayesian approach, we attempted to replicate a key study in the field of unconscious cognition. As in Vorberg et al. (2003), we asked participants to discriminate the direction of a prime or a mask. In an initial session, we first determined whether we could replicate the data pattern in Vorberg et al. (2003). Next, we introduced several key changes to determine whether these could explain the original data pattern they observed. The overall design was the same as Experiment 1 in Vorberg et al. (2003), but participants were thoroughly instructed and underwent extensive practice. We also introduced a long SOA in which the relevant feature of the prime stimulus was very clear to reduce the overall perceived difficulty of the experiment and to prevent participants from giving up. Further, the analyses were performed at the single subject level in a Bayesian framework (see the Introduction for a list of the main changes). First, we tested for unconscious priming using the double *t-*test approach to see if we could replicate the results by Vorberg et al. (2003). Then, we recoded the RTs of the mask discrimination task using the median-split technique to directly compare prime awareness and priming.

The data pattern from the initial session mostly replicated the original results by Vorberg et al. (2003). In our modified version of the task, however, we obtained markedly different results. When testing priming and prime awareness separately, using the so-called double *t-*test approach, we observed unconscious priming, although limited to some participants and some SOAs. In contrast to Vorberg et al. (2003), prime discrimination performance at the group level was overall above chance and increased at longer SOAs, similar to the priming effect, albeit with smaller effect sizes. At the single subject level, most participants were able to discriminate the direction of the prime across most SOAs, plausibly due to the extensive training introduced in our modified version of the task. Note however, that some participants seemed to be able to discriminate the direction of the prime at longer SOAs from the very beginning, without the need for practice or extensive instructions. Given the small sample size in our study and in Vorberg et al. (2003), it is possible that the observers in our samples differed in their level of perceptual sensitivity for metacontrast masked stimuli and/or in their metacontrast masking function, as some observers have been shown to follow a Type A (monotonously increasing) masking function, whereas others show a Type B (U-shaped) masking function (Albrecht et al. 2010, Albrecht and Mattler 2012).

Another possibility is that including the 300 ms SOA not only helped keep participants motivated, but also influenced participants’ strategy or decision criterion (Lockhead 2004, King and Dehaene 2014, Mill et al. 2014). We tested this possibility by comparing the decision criterion between session 1, which did not include the 300 ms SOA, and session 2, which included the 300 ms SOA. This analysis provided strong evidence for no difference in decision criterion, suggesting that the wider SOA range did not measurably alter response criterion. However, this analysis cannot rule out possible effects on motivation, engagement, or other aspects of task strategy.

However, the discrepancy in results might also be due to a more fundamental difference between studies. First, although the timing of the stimuli and SOAs were different in our study compared to Vorberg et al. (2003), they were well within the time range of the original study (14–70 ms in the original study and 12.5–75 ms in our study). Given the duration of the prime in our study was shorter (12.5 ms) compared to the original study (14 ms), it is unlikely that differences in timing could explain the increased performance in the prime discrimination task we observed in our study. Second, in our study, we used a liquid-crystal display (LCD) to present the stimuli, unlike the cathode ray tube (CRT) monitor used in the original study. CRT monitors are characterized by superior response time compared to LCD monitors (Lagroix et al. 2012), meaning that the observed duration of stimuli presented on CRT monitors should be in principle closer to the intended duration. It is possible, e.g. that the stimuli in our experiment were presented for longer than intended due to the relatively sluggish response time of LCD pixels. Although we cannot completely rule out this possibility, a number of studies have shown similar performance in perceptual tasks across CRT and LCD monitors (Kihara et al. 2010, Lagroix et al. 2012, Bognár et al. 2016). Crucially, one of them being in a masked prime discrimination (Rohr and Wagner 2020), and one of them using metacontrast masking (Kihara et al. 2010). Therefore, although not impossible, it is unlikely that a difference in the display technology could fully explain the increased prime awareness we find in our study.

Overall, it is not possible to conclude with certainty which of the changes we implemented in our version of the experiment resulted in an increased prime discrimination performance. While thorough instructions and practice are a plausible contributor, some participants were above chance at prime discrimination already in the first session (before comprehensive instructions and practice). Instead, it is plausible that several of the changes we introduced contributed to improved performance across participants.

When focusing on the single subject results, another interesting observation is that the SOA-priming profile was highly consistent across subjects, whereas prime awareness was much more variable. For example, some participants showed above chance prime discrimination for all or most SOAs, whereas others performed at chance or near-chance at most SOAs. In stark contrast, all participants showed the same pattern of results in the mask discrimination task, no priming effect at the shortest SOA, with a gradual increase in priming at longer SOAs. The variability in prime awareness aligns with previous claims that awareness measures are often noisy and unreliable (Vadillo et al. 2022, Hernández-Gutiérrez et al. 2025). However, in our case, the estimations we obtained for each subject should be fairly precise, given that each participant was trained before completing five or six one-hour sessions, summing up to ~500 trials per SOA. Accordingly, we show that the internal consistency of prime discrimination responses was quite high across all but the 300 ms SOA. Therefore, the variability we observe is unlikely to reflect measurement noise alone and is at least partly consistent with genuine individual differences in perceptual sensitivity (we discuss the potential sources for this variability further below).

To overcome the problems of the double *t-*test approach, we recoded the rts in the mask discrimination task as accuracy using the median-split technique and directly compared it with the prime discrimination performance (Meyen et al. 2022), which has also been referred to as sensitivity dissociation (Schmidt and Vorberg 2006). In this framework, the indirect effect is driven by conscious information that should also be present in the direct effect. Therefore, to explain an indirect task advantage, there must be an additional source of unconscious information not captured by the direct measure that the indirect measure capitalizes on. It is important to note that this inference relies on the assumption that the direct task is at least as sensitive as the indirect task to the relevant prime information (Schmidt and Vorberg 2006). In any case, this assumption is more permissive than other approaches that rely on awareness measures being exhaustively sensitive and on the zero-awareness criterion (Schmidt 2008).

Note that dichotomizing continuous measures inevitably entails some information loss (Cohen 1983), a critique that has been raised over the median-split technique in priming literature (Zerweck et al. 2021, Meyen et al. 2022, Stein et al. 2024, Hernández-Gutiérrez et al. 2025). However, in our data, raw and recoded priming values are highly correlated at both the subject and group level, and previous work has similarly reported high fidelity between raw and transformed priming effects (Zerweck et al. 2021, Meyen et al. 2022, Stein et al. 2024, Hernández-Gutiérrez et al. 2025). Additionally, the prime discrimination task itself requires participants to give binary responses, presumably based on a continuous perceptual experience. For the present direct-comparison approach, this makes it necessary to transform the priming RT measure into a binary response format so that the two tasks can be compared on a common scale (Meyen et al. 2022).

Using this approach, we found an indirect task advantage (priming effect larger than prime discrimination performance) across all participants in at least one SOA. Importantly, the same participants who showed unconscious priming when using the double *t-*test approach also showed an indirect task advantage in at least one SOA. Because, unlike the double *t-*test approach, the direct comparison provides a valid test of an indirect task advantage, these results strengthen the evidence for unconscious priming under the assumption that the direct task is at least as sensitive as the indirect task. This evidence was nevertheless present only for some participants and some SOAs. Note again that concluding unconscious priming based on an indirect task advantage relies on the assumption that the direct task is at least as sensitive as the indirect task, and that this assumption is still debated (Schmidt and Vorberg 2006, Stockart et al. 2025).

The source of the interindividual variance in perceptual sensitivity for prime discrimination remains unclear in our study. One possibility is that there are individual differences in the sensitivity to masked stimuli, resulting in some participants having less conscious information available than others, while the observed priming effect is not influenced by such differences. Another possibility is that—despite the training we provided—participants differ in the degree to which they can convert task instructions for prime discrimination into the correct actions despite having equal conscious experiences. Notably, the task to extract the direction of the prime is not an easy one, given the rapid sequence of stimuli that appear, which may be more confusing to some than to others, despite having similar perceptual experiences. Thus, some participants may rely on specific local features of the masked stimulus to perform the task which others are not able to use as effectively (Albrecht et al. 2010, Albrecht and Mattler 2012). For example, even if the outline of the arrow is not fully visible, one might use cues such as the perceived centre of mass of the prime: an arrow pointing right should have more mass towards the right. Such features are, in principle, available to all participants, but only some may be able to exploit them effectively. In line with this interpretation, previous research in a metacontrast paradigm has shown that above chance performance is associated with different perceptual cues (perceived motion or after-images) at short and long SOAs (Albrecht and Mattler 2012). In this study, participants that performed poorly when discriminating stimuli masked with a metacontrast mask reported the occurrence of perceptual cues with similar frequency to participants with better performance, but nevertheless failed at using the perceptual cues to successfully perform the task at any SOA.

However, it is also possible that training did not influence the ability of participants to translate local perceptual cues into objective performance but that participants learned to ‘see’ the stimuli. A number of studies have reported improved performance in tasks using metacontrast masking (for instance, Hernandez and Lefton 1977, Hogben and Di Lollo 1984, Schwiedrzik et al. 2009, 2011). In one of them, participants became objectively aware of the stimuli after being trained at a prime-mask SOA for which their initial performance was at chance (Schwiedrzik et al. 2009, 2011). These results, nevertheless, contrast with the original results by Vorberg et al. (2003), in which participants completed more than 3000 trials with trial feedback and still showed chance performance across all SOAs. A potential explanation for the lack of improvement in the study by Vorberg et al. (2003) is the delay between the mask offset and the response window, as subjects could have been briefly aware of the stimulus but quickly forgot (Schwiedrzik et al. 2009). However, we show in our study that performance improvement is possible with the same response delay. Altogether, it remains difficult to definitively settle whether training leads to the better use of the already available conscious information, and therefore to better performance, or whether participants learn to ‘see’ stimuli that were not perceived before the training.

To conclude, our findings provide evidence for unconscious priming at the single-subject level using the direct-comparison approach, while also emphasizing that this conclusion depends on the assumption that the direct task is at least as sensitive as the indirect task. Our study also highlights the importance of providing clear instructions and sufficient training to appropriately assess awareness; otherwise, apparent lack of awareness may partially reflect disengagement and/or an inability to convert perceptual experiences into behavioural responses. However, even with such measures and procedures in place, individual differences may pose a problem when concluding lack of awareness across participants, as interindividual differences in performance on the awareness measure may be confounded by differences in the ability to appropriately respond to task demands.

## Supplementary Material

si_niag052

## Data Availability

Data and analysis scripts are available at https://doi.org/10.17605/OSF.IO/3M6FU.
