# Supplementary material for: Evidence for unconscious priming using a Bayesian single-subject approach
Source: Neurosci Conscious. 2026 Aug 20;2026(1):niag052. doi: 10.1093/nc/niag052 (PMC13492662; doi:10.1093/nc/niag052)
Supplement: Supplementary file 1 [file si_niag052.pdf]

# Supporting Information for *Evidence for unconscious priming using a Bayesian single-subject approach*

Nicolás Sánchez-Fuenzalida[1,2,✉], Simon van Gaal[3,4,1,2], Zazie van den Hurk[1,2], Timo Stein[3,4], and Johannes J. Fahrenfort[1,2,3,4]

[1] Department of Applied and Experimental Psychology, Free University Amsterdam; [2] Institute for Brain and Behavior Amsterdam, Free University Amsterdam; [3] Department of Psychology, University of Amsterdam; [4] Amsterdam Brain & Cognition, University of Amsterdam

✉ Correspondence: Nicolás Sánchez-Fuenzalida <nicolas.carvajalsanchez@unibas.ch>

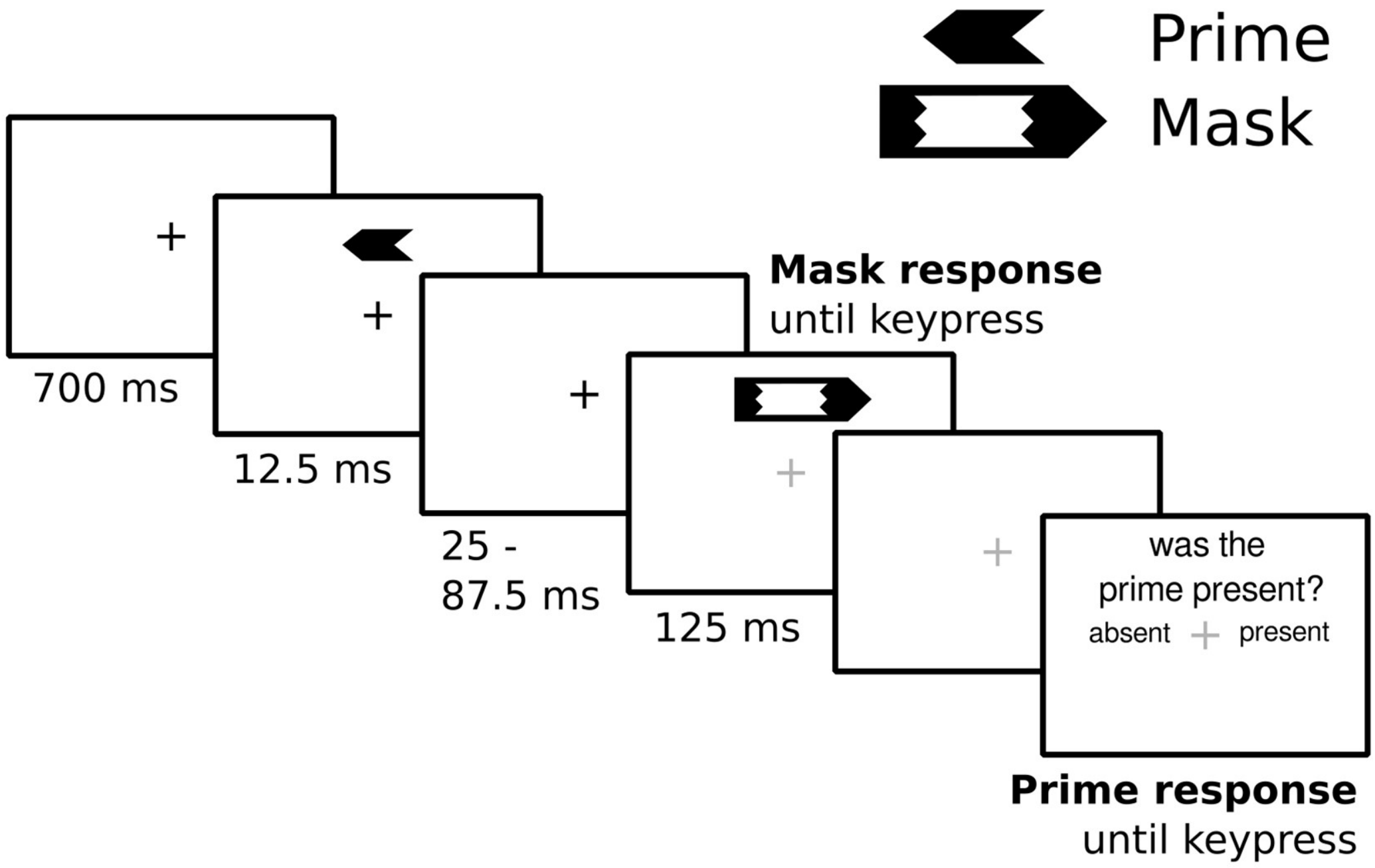


*Figure S1:* ***Combined mask discrimination and prime detection task.*** *Trials started with a fixation period of 700 ms, followed by the prime (12.5 ms), followed by a variable SOA period (12.5 - 75 ms or 300 ms), followed by the mask (125 ms). The fixation turned light grey as soon as the mask was presented to indicate participants that they could answer. The fixation remained on screen until a response was registered. 250 ms after the response participants were prompted to indicate whether the prime was presented (until keypress)*

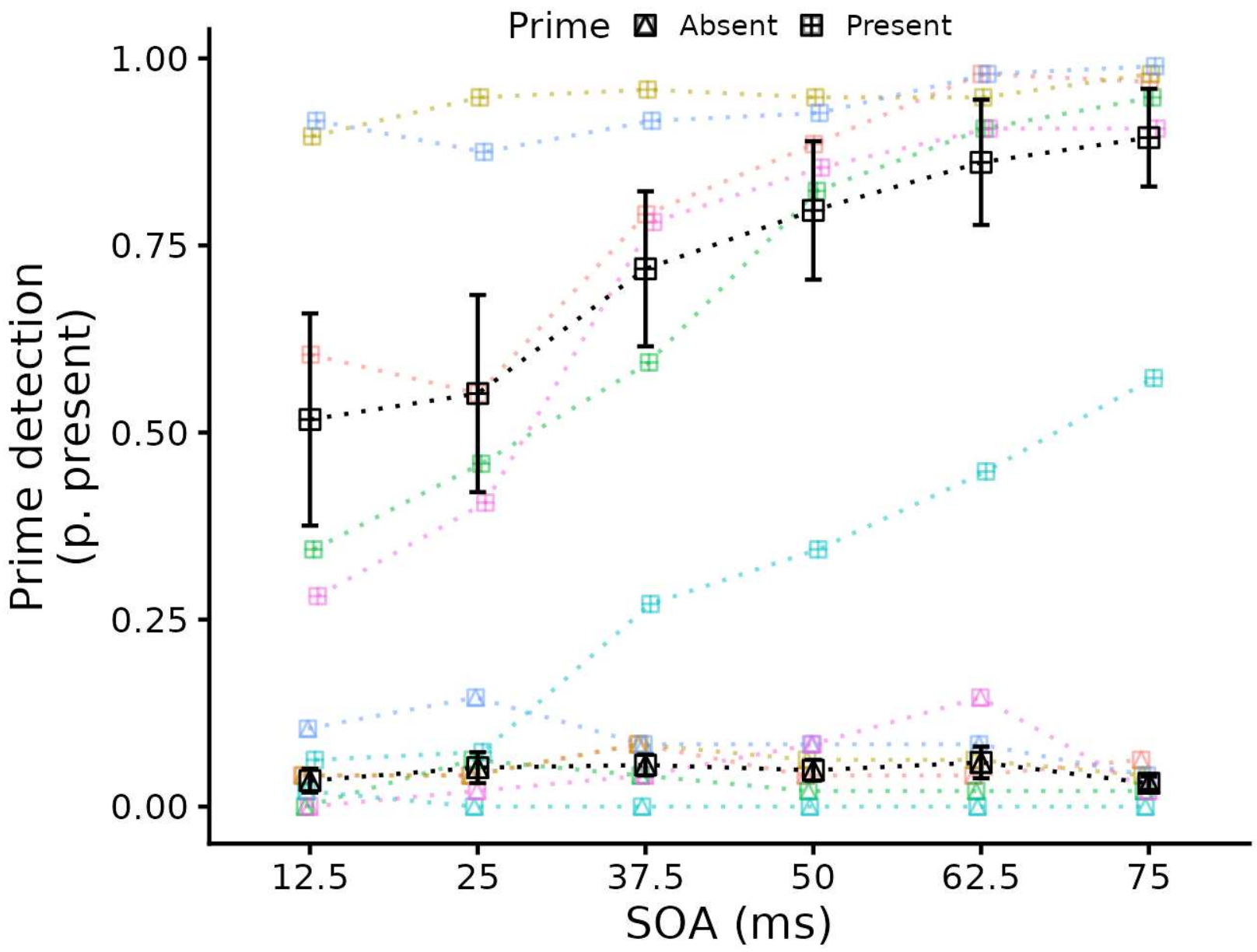


*Figure S2:* ***Control Experiment Prime detection results.*** *Subject- (colour) and group-level detection results. Averages indicate the proportion of present responses on present trials (square with cross) and absent trials (square with triangle). Error bars indicate the SEM*

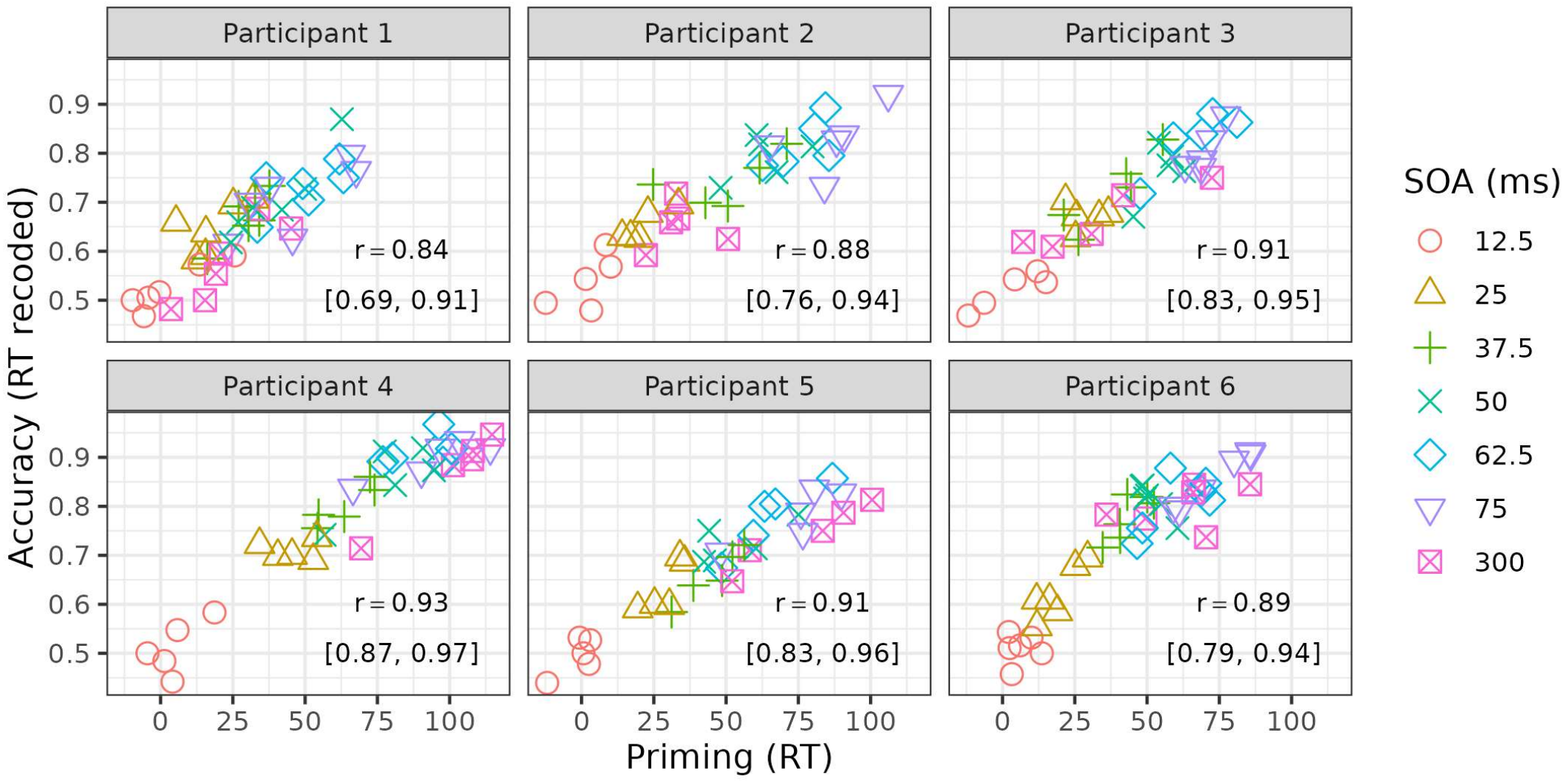


*Figure S3:* ***Correlation between raw and transformed priming effect****. Correlation between raw priming effect (incongruent minus congruent mask discrimination reaction time) and priming RT-recoded as accuracy plotted separately for each participant. Each SOA is depicted as a different shape and colour. Different sessions of the same SOA share shape and colour. The median correlation (r) plus 95% credible interval was estimated across sessions and SOAs separately for each participant. To estimate the correlation coefficient 10,000 posterior samples were drawn from the correlation performed with correlationBF function {BayesFactor package}.*

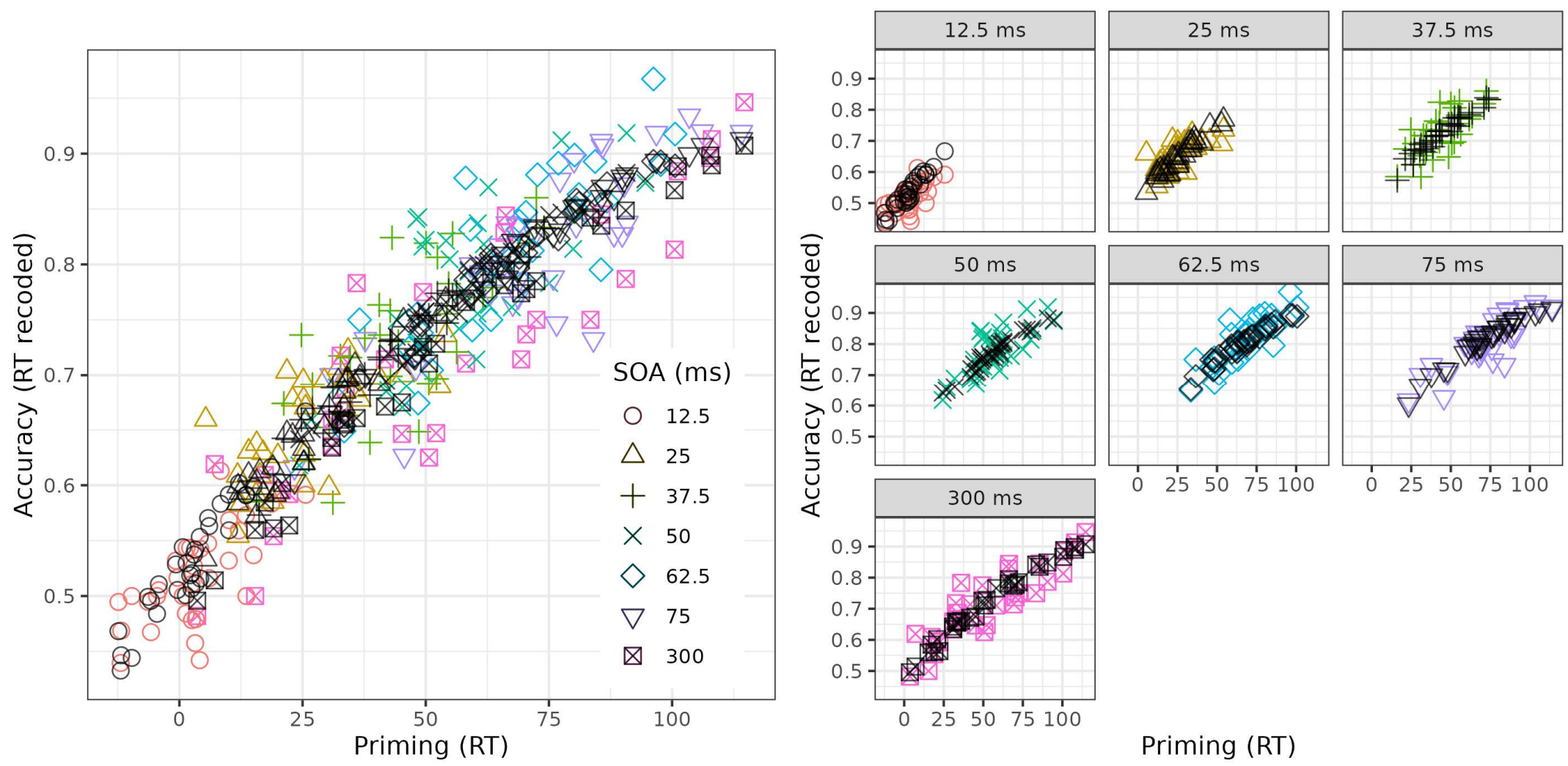


*Figure S4:* ***Regression between raw and transformed priming effect****. Raw priming effect (incongruent minus congruent mask discrimination reaction time) and priming RT-recoded as accuracy are plotted as in Sup. Figure S3, but the data of all participants, sessions and SOAs are plotted together (left-panel) or separated by SOA (right-panel). Black shapes are the model-predicted raw and recoded priming effects.*

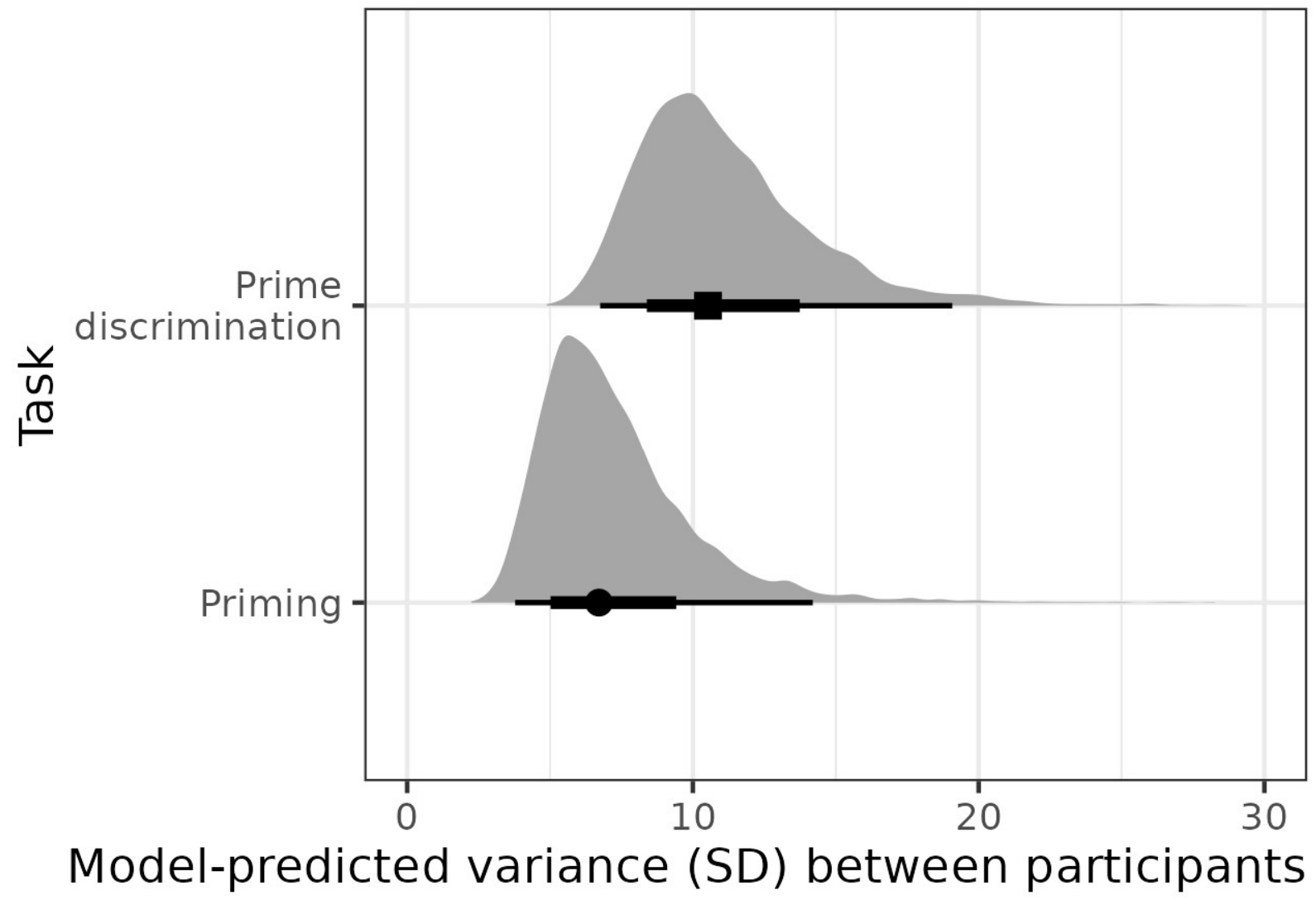


*Figure S5:* ***Model-predicted variance between participants per task****. Posterior distribution of the variance across participants separately for prime and mask (RT-recoded) discrimination task. Dot and square depict the mean across participants model-predicted SD along with the 66% (thick line) and 95% (thin line) credible interval.*

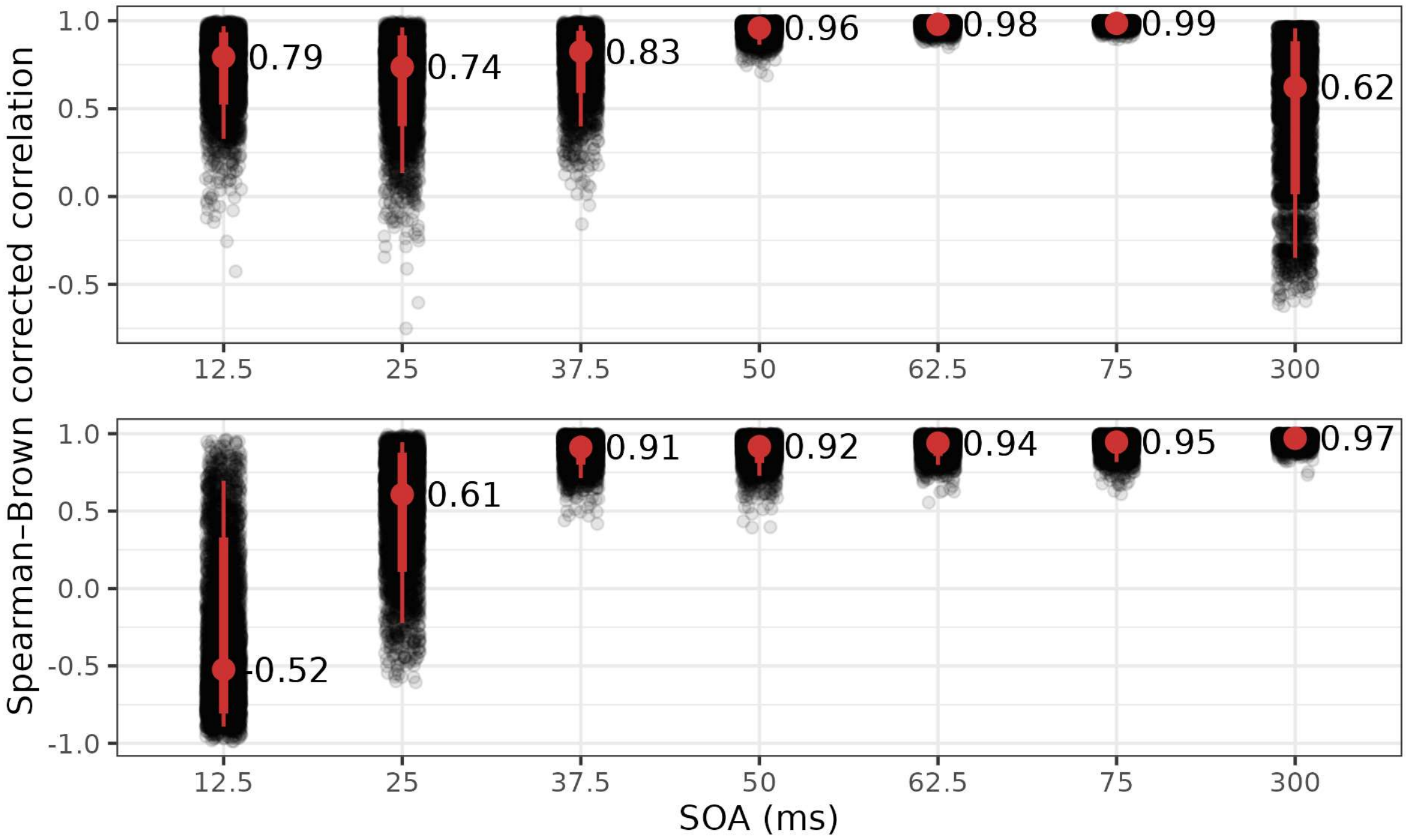


*Figure S6:* ***Prime and mask discrimination internal consistency****. Median (red dot) permutation-based split-half reliability using 5000 random splits. Each black dot represents the Spearman-Brown corrected correlation across participants separately for each SOA and task. Thick (66%) and thin (95%) red lines indicate credible intervals.*

*Table S1: RT trimming robustness check for priming results of the control experiment analysis (compare with Figure 2B).*

| SOA | stat | 0.5% | 1% | 2% |
|---|---|---|---|---|
| 12.5 ms | $BF_{10}$ | 0.003 | 0.002 | 0.003 |
| | *d* | 0.13 | 0.11 | 0.14 |
| 25 ms | $BF_{10}$ | $10^{29}$ | $10^{15}$ | $10^{97}$ |
| | *d* | 0.75 | 0.75 | 0.75 |
| 37.5 ms | $BF_{10}$ | $10^{19}$ | $>10^{40}$ | $10^{53}$ |
| | *d* | 1.01 | 1.01 | 1.04 |
| 50 ms | $BF_{10}$ | $10^{22}$ | $10^{37}$ | $10^{55}$ |
| | *d* | 1.08 | 1.08 | 1.11 |
| 62.5 ms | $BF_{10}$ | $10^{286}$ | $>10^{40}$ | $10^{139}$ |
| | *d* | 1.35 | 1.27 | 1.26 |
| 75 ms | $BF_{10}$ | $10^{17}$ | $10^{17}$ | $10^{17}$ |
| | *d* | 1.86 | 1.86 | 1.87 |

*Table S2: RT trimming robustness check for priming results of the Experiment 1 group-level results (compare with Figure 1B and C).*

| SOA | stat | 0.5% | 1% | 2% |
|---|---|---|---|---|
| 12.5 ms | $BF_{10}$ | 0.019 | 0.009 | $10^{4}$ |
| | *d* | 0.12 | 0.1 | 0.18 |
| 25 ms | $BF_{10}$ | $10^{18}$ | $10^{19}$ | $10^{13}$ |
| | *d* | 0.87 | 0.8 | 0.87 |
| 37.5 ms | $BF_{10}$ | $10^{14}$ | $10^{13}$ | $10^{13}$ |
| | *d* | 1.39 | 1.4 | 1.33 |
| 50 ms | $BF_{10}$ | $10^{13}$ | $10^{13}$ | $10^{14}$ |
| | *d* | 1.74 | 1.7 | 1.67 |
| 62.5 ms | $BF_{10}$ | $10^{13}$ | $10^{13}$ | $10^{13}$ |
| | *d* | 1.99 | 2 | 1.98 |
| 75 ms | $BF_{10}$ | $10^{14}$ | $10^{13}$ | $10^{13}$ |
| | *d* | 1.99 | 2 | 1.95 |
| 300 ms | $BF_{10}$ | $10^{14}$ | $10^{14}$ | $10^{16}$ |
| | *d* | 1.43 | 1.3 | 1.3 |

*Table S3: RT trimming robustness check for priming results of the Experiment 1 subject-level results (compare with Figure 4 right-side).*

| Pp | SOA | stat | 0.5% | 1% | 2% | Pp | SOA | stat | 0.5% | 1% | 2% | Pp | SOA | stat | 0.5% | 1% | 2% |
|---|---|---|---|---|---|---|---|---|---|---|---|---|---|---|---|---|---|
| 1 | 12.5 | $BF_{10}$ | 0.001 | 0.001 | 0.001 | 3 | 12.5 | $BF_{10}$ | 0.001 | 0.001 | 0.006 | 5 | 12.5 | $BF_{10}$ | 0.001 | 0.001 | 0.001 |
| | | *d* | 0.1 | 0.1 | 0.1 | | | *d* | 0.1 | 0.1 | 0.2 | | | *d* | 0 | 0 | 0.1 |
| | 25 | $BF_{10}$ | $10^{14}$ | $10^{14}$ | $10^{114}$ | | 25 | $BF_{10}$ | $10^{16}$ | $10^{24}$ | $10^{18}$ | | 25 | $BF_{10}$ | $10^{13}$ | $>10^{40}$ | $10^{15}$ |
| | | *d* | 0.7 | 0.7 | 0.7 | | | *d* | 0.7 | 0.7 | 0.7 | | | *d* | 0.7 | 0.7 | 0.8 |
| | 37.5 | $BF_{10}$ | $10^{65}$ | $>10^{40}$ | $10^{34}$ | | 37.5 | $BF_{10}$ | $10^{35}$ | $>10^{40}$ | $10^{142}$ | | 37.5 | $BF_{10}$ | $10^{37}$ | $10^{139}$ | $10^{64}$ |
| | | *d* | 1 | 1 | 0.9 | | | *d* | 1 | 0.9 | 1 | | | *d* | 0.9 | 0.9 | 1 |
| | 50 | $BF_{10}$ | $10^{17}$ | $10^{18}$ | $10^{17}$ | | 50 | $BF_{10}$ | $10^{15}$ | $10^{13}$ | $10^{20}$ | | 50 | $BF_{10}$ | $10^{16}$ | $>10^{40}$ | $10^{18}$ |
| | | *d* | 1.1 | 1 | 1 | | | *d* | 1.6 | 1.5 | 1.5 | | | *d* | 1.4 | 1.3 | 1.3 |
| | 62.5 | $BF_{10}$ | $10^{24}$ | $>10^{40}$ | $10^{23}$ | | 62.5 | $BF_{10}$ | $10^{17}$ | $>10^{40}$ | $10^{13}$ | | 62.5 | $BF_{10}$ | $10^{16}$ | $10^{40}$ | $10^{17}$ |
| | | *d* | 1.1 | 1.1 | 1.1 | | | *d* | 2.2 | 2.2 | 2.1 | | | *d* | 1.5 | 1.5 | 1.5 |
| | 75 | $BF_{10}$ | $10^{21}$ | $10^{14}$ | $10^{26}$ | | 75 | $BF_{10}$ | $10^{14}$ | $>10^{40}$ | $10^{21}$ | | 75 | $BF_{10}$ | $10^{25}$ | $10^{15}$ | $10^{15}$ |
| | | *d* | 1.1 | 1.1 | 1.1 | | | *d* | 1.8 | 1.8 | 1.8 | | | *d* | 1.7 | 1.7 | 1.7 |
| | 300 | $BF_{10}$ | $10^{17}$ | $10^{14}$ | $10^{15}$ | | 300 | $BF_{10}$ | $10^{50}$ | $10^{16}$ | $10^{16}$ | | 300 | $BF_{10}$ | $10^{20}$ | $10^{24}$ | $10^{164}$ |
| | | *d* | 0.7 | 0.5 | 0.6 | | | *d* | 0.8 | 0.7 | 0.7 | | | *d* | 1.3 | 1.3 | 1.3 |
| 2 | 12.5 | $BF_{10}$ | 0.005 | 0.004 | 0.016 | 4 | 12.5 | $BF_{10}$ | 0.002 | 0.001 | 0.015 | 6 | 12.5 | $BF_{10}$ | 0.003 | 0.003 | 0.007 |
| | | *d* | 0.2 | 0.2 | 0.3 | | | *d* | 0.2 | 0.1 | 0.3 | | | *d* | 0.2 | 0.2 | 0.2 |
| | 25 | $BF_{10}$ | $10^{24}$ | $>10^{40}$ | $10^{17}$ | | 25 | $BF_{10}$ | $10^{106}$ | $>10^{40}$ | $10^{17}$ | | 25 | $BF_{10}$ | $10^{13}$ | $10^{17}$ | $10^{22}$ |
| | | *d* | 0.9 | 0.9 | 0.9 | | | *d* | 1.4 | 1.3 | 1.4 | | | *d* | 0.7 | 0.6 | 0.7 |
| | 37.5 | $BF_{10}$ | $10^{15}$ | $10^{16}$ | $10^{45}$ | | 37.5 | $BF_{10}$ | $10^{37}$ | $10^{16}$ | $10^{17}$ | | 37.5 | $BF_{10}$ | $10^{15}$ | $10^{22}$ | $10^{16}$ |
| | | *d* | 1.5 | 1.5 | 1.5 | | | *d* | 2.3 | 2.2 | 2.2 | | | *d* | 1.7 | 1.6 | 1.6 |
| | 50 | $BF_{10}$ | $10^{16}$ | $10^{17}$ | $10^{19}$ | | 50 | $BF_{10}$ | $10^{14}$ | $10^{13}$ | $10^{14}$ | | 50 | $BF_{10}$ | $10^{13}$ | $10^{13}$ | $10^{13}$ |
| | | *d* | 1.9 | 1.9 | 1.8 | | | *d* | 2.8 | 2.7 | 2.6 | | | *d* | 2.2 | 2.1 | 2 |
| | 62.5 | $BF_{10}$ | $10^{13}$ | $>10^{40}$ | $10^{16}$ | | 62.5 | $BF_{10}$ | $10^{14}$ | $10^{16}$ | $10^{17}$ | | 62.5 | $BF_{10}$ | $10^{15}$ | $10^{14}$ | $10^{16}$ |
| | | *d* | 2.3 | 2.3 | 2.2 | | | *d* | 3.1 | 3.1 | 3.1 | | | *d* | 2 | 2 | 2 |
| | 75 | $BF_{10}$ | $10^{18}$ | $10^{17}$ | $10^{15}$ | | 75 | $BF_{10}$ | $10^{14}$ | $>10^{40}$ | $10^{14}$ | | 75 | $BF_{10}$ | $10^{14}$ | $10^{15}$ | $10^{14}$ |
| | | *d* | 2.2 | 2.2 | 2.1 | | | *d* | 2.8 | 2.7 | 2.7 | | | *d* | 2.3 | 2.2 | 2.2 |
| | 300 | $BF_{10}$ | $10^{43}$ | $10^{17}$ | $10^{46}$ | | 300 | $BF_{10}$ | $10^{14}$ | $10^{13}$ | $10^{17}$ | | 300 | $BF_{10}$ | $10^{15}$ | $10^{13}$ | $10^{17}$ |
| | | *d* | 1.3 | 1.2 | 1.2 | | | *d* | 3.6 | 3.5 | 3.4 | | | *d* | 2.3 | 2.2 | 2.1 |

*Table S4: RT trimming robustness check for the direct comparison results of the Experiment 1 subject-level results (compare with Figure 5A).*

| Pp | SOA | stat | 0.5% | 1% | 2% | Pp | SOA | stat | 0.5% | 1% | 2% | Pp | SOA | stat | 0.5% | 1% | 2% |
|---|---|---|---|---|---|---|---|---|---|---|---|---|---|---|---|---|---|
| 1 | 12.5 | $BF_{10}$ | 0.185 | 0.268 | 0.299 | 3 | 12.5 | $BF_{10}$ | 0.03 | 0.03 | 0.027 | 5 | 12.5 | $BF_{10}$ | 3.45 | 6.07 | 1.45 |
| | | *d* | -0.1 | -0.1 | -0.1 | | | *d* | 0 | 0 | 0 | | | *d* | -0.2 | -0.2 | -0.2 |
| | 25 | $BF_{10}$ | 0.235 | 0.182 | 0.896 | | 25 | $BF_{10}$ | $10^{19}$ | $10^{17}$ | $10^{15}$ | | 25 | $BF_{10}$ | 4.04 | 4.02 | 52 |
| | | *d* | 0.1 | 0.1 | 0.2 | | | *d* | 0.4 | 0.4 | 0.4 | | | *d* | 0.2 | 0.2 | 0.3 |
| | 37.5 | $BF_{10}$ | 0.716 | 0.756 | 1.35 | | 37.5 | $BF_{10}$ | $10^{15}$ | $10^{15}$ | $10^{18}$ | | 37.5 | $BF_{10}$ | 76 | $10^{6}$ | $10^{9}$ |
| | | *d* | 0.2 | 0.2 | 0.2 | | | *d* | 0.5 | 0.5 | 0.5 | | | *d* | 0.3 | 0.3 | 0.3 |
| | 50 | $BF_{10}$ | 4.69 | 6.43 | 19 | | 50 | $BF_{10}$ | $10^{23}$ | $10^{15}$ | $10^{15}$ | | 50 | $BF_{10}$ | $10^{15}$ | $>10^{40}$ | $10^{18}$ |
| | | *d* | 0.2 | 0.2 | 0.2 | | | *d* | 0.6 | 0.6 | 0.6 | | | *d* | 0.4 | 0.4 | 0.5 |
| | 62.5 | $BF_{10}$ | $10^{5}$ | $10^{3}$ | $10^{5}$ | | 62.5 | $BF_{10}$ | $10^{17}$ | $10^{16}$ | $10^{17}$ | | 62.5 | $BF_{10}$ | $10^{15}$ | $10^{15}$ | $10^{15}$ |
| | | *d* | 0.3 | 0.3 | 0.3 | | | *d* | 0.7 | 0.7 | 0.8 | | | *d* | 0.6 | 0.6 | 0.6 |
| | 75 | $BF_{10}$ | 0.241 | 0.249 | 0.472 | | 75 | $BF_{10}$ | $10^{17}$ | $10^{15}$ | $10^{16}$ | | 75 | $BF_{10}$ | $10^{17}$ | $10^{16}$ | $10^{16}$ |
| | | *d* | 0.2 | 0.2 | 0.2 | | | *d* | 0.6 | 0.6 | 0.6 | | | *d* | 0.5 | 0.5 | 0.5 |
| | 300 | $BF_{10}$ | $10^{31}$ | $10^{16}$ | $10^{24}$ | | 300 | $BF_{10}$ | $10^{24}$ | $10^{37}$ | $10^{19}$ | | 300 | $BF_{10}$ | $10^{16}$ | $10^{19}$ | $10^{16}$ |
| | | *d* | -3.4 | -3.4 | -3.4 | | | *d* | -4.2 | -4.2 | -4.2 | | | *d* | -1.8 | -1.9 | -1.9 |
| 2 | 12.5 | $BF_{10}$ | 0.032 | 0.028 | 0.037 | 4 | 12.5 | $BF_{10}$ | 0.028 | 0.026 | 0.05 | 6 | 12.5 | $BF_{10}$ | 0.024 | 0.024 | 0.029 |
| | | *d* | 0 | 0 | 0.1 | | | *d* | 0 | 0 | 0.1 | | | *d* | 0 | 0 | 0 |
| | 25 | $BF_{10}$ | 0.102 | 0.095 | 0.238 | | 25 | $BF_{10}$ | $10^{16}$ | $>10^{40}$ | $10^{15}$ | | 25 | $BF_{10}$ | 0.98 | 0.541 | 2.48 |
| | | *d* | 0.1 | 0.1 | 0.2 | | | *d* | 0.5 | 0.5 | 0.5 | | | *d* | 0.2 | 0.2 | 0.2 |
| | 37.5 | $BF_{10}$ | 48 | 12 | 27 | | 37.5 | $BF_{10}$ | $10^{16}$ | $10^{15}$ | $10^{16}$ | | 37.5 | $BF_{10}$ | $10^{15}$ | $>10^{40}$ | $10^{19}$ |
| | | *d* | 0.3 | 0.3 | 0.3 | | | *d* | 0.6 | 0.6 | 0.6 | | | *d* | 0.5 | 0.5 | 0.6 |
| | 50 | $BF_{10}$ | 0.264 | 0.252 | 0.433 | | 50 | $BF_{10}$ | $10^{15}$ | $10^{15}$ | $10^{17}$ | | 50 | $BF_{10}$ | $10^{2}$ | 19 | $10^{2}$ |
| | | *d* | 0.2 | 0.2 | 0.2 | | | *d* | 0.6 | 0.6 | 0.7 | | | *d* | 0.3 | 0.3 | 0.3 |
| | 62.5 | $BF_{10}$ | 0.037 | 0.041 | 0.039 | | 62.5 | $BF_{10}$ | $10^{15}$ | $10^{16}$ | $10^{17}$ | | 62.5 | $BF_{10}$ | 0.045 | 0.038 | 0.083 |
| | | *d* | 0 | 0 | 0 | | | *d* | 0.8 | 0.9 | 0.9 | | | *d* | 0.1 | 0.1 | 0.1 |
| | 75 | $BF_{10}$ | 59 | $10^{2}$ | $10^{2}$ | | 75 | $BF_{10}$ | $10^{17}$ | $>10^{40}$ | $10^{15}$ | | 75 | $BF_{10}$ | 0.05 | 0.045 | 0.061 |
| | | *d* | -0.4 | -0.5 | -0.4 | | | *d* | 0.7 | 0.6 | 0.7 | | | *d* | 0.1 | 0.1 | 0.1 |
| | 300 | $BF_{10}$ | $10^{23}$ | $10^{23}$ | $10^{37}$ | | 300 | $BF_{10}$ | $10^{23}$ | $10^{16}$ | $10^{23}$ | | 300 | $BF_{10}$ | $10^{17}$ | $10^{16}$ | $10^{16}$ |
| | | *d* | -2.3 | -2.3 | -2.3 | | | *d* | -2 | -2.1 | -2 | | | *d* | -1.8 | -1.9 | -1.8 |

*Table S5: RT trimming robustness check for the direct comparison results of the Experiment 1 group-level results (compare with Figure 5B).*

| SOA | stat | 0.5% | 1% | 2% |
|---|---|---|---|---|
| 12.5 ms | $BF_{10}$ | 0.056 | 0.083 | 0.018 |
| | *d* | -0.1 | -0.1 | 0 |
| 25 ms | $BF_{10}$ | $10^{17}$ | $10^{121}$ | $10^{16}$ |
| | *d* | 0.2 | 0.2 | 0.3 |
| 37.5 ms | $BF_{10}$ | $10^{16}$ | $10^{16}$ | $10^{16}$ |
| | *d* | 0.4 | 0.4 | 0.4 |
| 50 ms | $BF_{10}$ | $10^{15}$ | $10^{16}$ | $10^{18}$ |
| | *d* | 0.4 | 0.4 | 0.4 |
| 62.5 ms | $BF_{10}$ | $10^{15}$ | $10^{15}$ | $10^{15}$ |
| | *d* | 0.4 | 0.4 | 0.4 |
| 75 ms | $BF_{10}$ | $10^{15}$ | $10^{134}$ | $10^{20}$ |
| | *d* | 0.3 | 0.3 | 0.3 |
| 300 ms | $BF_{10}$ | $10^{17}$ | $10^{20}$ | $10^{19}$ |
| | *d* | -2.3 | -2.4 | -2.4 |

*Table S6: Prime discrimination Bayes Factors ($BF_{10}$) and Cohen's d values for each participant (P).*

| P | stat | 12.5 ms | 25 ms | 37.5 ms | 50 ms | 62.5 ms | 75 ms | 300 ms |
|---|---|---|---|---|---|---|---|---|
| 1 | $BF_{10}$ | $10^{6}$ | $10^{7}$ | $10^{15}$ | $10^{15}$ | $10^{11}$ | $10^{17}$ | $10^{16}$ |
| 1 | *d* | 0.4 | 0.4 | 0.5 | 0.5 | 0.4 | 0.7 | 8.7 |
| 2 | $BF_{10}$ | 0.071 | $10^{4}$ | $>10^{40}$ | $10^{15}$ | $10^{19}$ | $10^{19}$ | $10^{16}$ |
| 2 | *d* | 0.1 | 0.5 | 0.6 | 1.2 | 1.9 | 2.8 | 6.9 |
| 3 | $BF_{10}$ | 0.141 | 0.04 | 0.081 | 0.085 | 3.92 | 97 | $>10^{40}$ |
| 3 | *d* | 0.2 | 0.0 | 0.1 | 0.1 | 0.3 | 0.4 | 8.9 |
| 4 | $BF_{10}$ | 0.038 | 0.047 | $10^{4}$ | $10^{20}$ | $10^{15}$ | $10^{15}$ | $10^{16}$ |
| 4 | *d* | 0.0 | 0.1 | 0.4 | 0.8 | 0.9 | 1.2 | 7.7 |
| 5 | $BF_{10}$ | $10^{10}$ | 0.095 | 0.05 | 0.438 | 1.1 | $10^{7}$ | $10^{16}$ |
| 5 | *d* | 0.5 | 0.1 | 0.1 | 0.2 | 0.3 | 0.5 | 6.6 |
| 6 | $BF_{10}$ | 0.034 | 0.414 | 88 | $10^{15}$ | $>10^{40}$ | $10^{15}$ | $10^{17}$ |
| 6 | *d* | 0.0 | 0.2 | 0.4 | 1.1 | 1.5 | 1.9 | 7.0 |

*Table S7: Mask discrimination Bayes Factors ($BF_{10}$) and Cohen's d values for each participant (P).*

| P | stat | 12.5 ms | 25 ms | 37.5 ms | 50 ms | 62.5 ms | 75 ms | 300 ms |
|---|---|---|---|---|---|---|---|---|
| 1 | $BF_{10}$ | 0.001 | $10^{14}$ | $>10^{40}$ | $10^{18}$ | $>10^{40}$ | $10^{14}$ | $10^{14}$ |
| 1 | *d* | 0.1 | 0.7 | 1.0 | 1.0 | 1.1 | 1.1 | 0.5 |
| 2 | $BF_{10}$ | 0.004 | $>10^{40}$ | $10^{16}$ | $10^{17}$ | $>10^{40}$ | $10^{17}$ | $10^{17}$ |
| 2 | *d* | 0.2 | 0.9 | 1.5 | 1.9 | 2.3 | 2.2 | 1.2 |
| 3 | $BF_{10}$ | 0.001 | $10^{24}$ | $>10^{40}$ | $10^{13}$ | $>10^{40}$ | $>10^{40}$ | $10^{16}$ |
| 3 | *d* | 0.1 | 0.7 | 0.9 | 1.5 | 2.2 | 1.8 | 0.7 |
| 4 | $BF_{10}$ | 0.001 | $>10^{40}$ | $10^{16}$ | $10^{13}$ | $10^{16}$ | $>10^{40}$ | $10^{13}$ |
| 4 | *d* | 0.1 | 1.3 | 2.2 | 2.7 | 3.1 | 2.7 | 3.5 |
| 5 | $BF_{10}$ | 0.001 | $>10^{40}$ | $10^{139}$ | $>10^{40}$ | $10^{40}$ | $10^{15}$ | $10^{24}$ |
| 5 | *d* | -0.0 | 0.7 | 0.9 | 1.3 | 1.5 | 1.7 | 1.3 |
| 6 | $BF_{10}$ | 0.003 | $10^{17}$ | $10^{22}$ | $10^{13}$ | $10^{14}$ | $10^{15}$ | $10^{13}$ |
| 6 | *d* | 0.2 | 0.6 | 1.6 | 2.1 | 2.0 | 2.2 | 2.2 |

*Table S8: Direct comparison Bayes Factors ($BF_{10}$) and Cohen's d values for each participant (P).*

| P | stat | 12.5 ms | 25 ms | 37.5 ms | 50 ms | 62.5 ms | 75 ms | 300 ms |
|---|---|---|---|---|---|---|---|---|
| 1 | $BF_{10}$ | 0.268 | 0.182 | 0.756 | 6.43 | $10^{3}$ | 0.249 | $10^{16}$ |
| 1 | *d* | -0.1 | 0.1 | 0.2 | 0.2 | 0.3 | 0.2 | -3.4 |
| 2 | $BF_{10}$ | 0.028 | 0.095 | 12 | 0.252 | 0.041 | $10^{2}$ | $10^{23}$ |
| 2 | *d* | 0.0 | 0.1 | 0.3 | 0.2 | -0.0 | -0.5 | -2.3 |
| 3 | $BF_{10}$ | 0.03 | $10^{17}$ | $10^{15}$ | $10^{15}$ | $10^{16}$ | $10^{15}$ | $10^{37}$ |
| 3 | *d* | -0.0 | 0.4 | 0.5 | 0.6 | 0.7 | 0.6 | -4.2 |
| 4 | $BF_{10}$ | 0.026 | $>10^{40}$ | $10^{15}$ | $10^{15}$ | $10^{16}$ | $>10^{40}$ | $10^{16}$ |
| 4 | *d* | 0.0 | 0.5 | 0.6 | 0.6 | 0.9 | 0.6 | -2.1 |
| 5 | $BF_{10}$ | 6.07 | 4.02 | $10^{6}$ | $>10^{40}$ | $10^{15}$ | $10^{16}$ | $10^{19}$ |
| 5 | *d* | -0.2 | 0.2 | 0.3 | 0.4 | 0.6 | 0.5 | -1.9 |
| 6 | $BF_{10}$ | 0.024 | 0.541 | $>10^{40}$ | 19 | 0.038 | 0.045 | $10^{16}$ |
| 6 | *d* | 0.0 | 0.2 | 0.5 | 0.3 | 0.1 | 0.1 | -1.9 |

## Supplementary Text S1: Alternative RT trimming robustness check

As the percentage of trials removed in the mask discrimination was not reported in the original manuscript, we opted for a conservative trimming of 1% on each tail (1% slowest and 1% fastest). However, this remains an arbitrary decision in our analysis. To test whether our results are robust against different RT trimming thresholds, we filtered the data of the mask discrimination task using half (0.5%) or double (2%) of the threshold we originally chose (1%) and repeated all mask discrimination analyses (see Sup. Tables S1, S2, S3, S4, and S5). When filtering the 0.5% slowest and fastest (1% total trimming), the results stayed the same except for one change in the direct comparison subject-level analysis, in which participant 2 at the 25 ms SOA went from strong to moderate evidence for no indirect task advantage. When filtering the 1% slowest and fastest (2% total trimming), the results were almost entirely the same. The biggest difference is that at the group level of our version of the experiment (Sup. Table S2), for the shortest SOA (12.5 ms), filtering 1% of each tail resulted in a change from strong evidence for the null to strong evidence for an effect, with the effect size going from 0.1 to 0.18. Additionally, there were four changes in the direct comparison subject-level analysis (Sup. Table S4) in which evidence went from moderate to strong for an indirect task advantage (participant 1, 50 ms SOA), from moderate to very strong for an indirect task advantage (participant 5, 25 ms SOA), from moderate to anecdotal evidence for an indirect task advantage (participant 5, 12.5 ms SOA), or from strong to moderate evidence for no indirect task advantage (participant 2, 25 ms SOA).

## Supplementary Text S2: Bernoulli probit regression to compare SDT decision criterion

There was strong to very strong evidence for no difference in decision criterion across SOAs between the first and second session for all participants (participant 1: $BF_{10}$ = 0.024; participant 2: $BF_{10}$ = 0.012; participant 3: $BF_{10}$ = 0.012; participant 4: $BF_{10}$ = 0.012; participant 5: $BF_{10}$ = 0.053; participant 6: $BF_{10}$ = 0.038).

To test whether SDT criterion differed between the first and second session, we fitted a separate Bernoulli probit regression model for each participant. The reported direction of the prime (*response*: left/right) was predicted by the direction of the prime stimulus (*stimulus*: left/right), *session*, and their interaction.

$$\text{response} \sim \text{stimulus} * \text{session}$$

For the intercept and fixed-effect regression coefficients, we used independent normal distributions centred on 0 with a standard deviation of 5 (Normal(0,5)), which are weakly informative. The model was fitted separately for each participant using the `brms` package in R with 4 chains of 4000 iterations each (1000 warmup).

SDT criterion was calculated from the model parameters following the regression-based SDT formulation (DeCarlo, 1998). With *stimulus* coded as 0/1, the intercept corresponds to the false-alarm rate on the probit scale, and the *stimulus* coefficient corresponds to d'.

Criterion was therefore calculated as the negative midpoint between the two response distributions. Bayes Factors were derived by testing the point hypothesis that the criterion difference between the first and second session was zero.

## Supplementary Text S3: Regression between raw and recoded priming effect

To test for the association between the recoded and raw priming effect, we fitted a Beta regression model to the average raw and recoded priming effect for each session and SOA. Raw priming effects (RT) and SOA values were z-scored before fitting the model. In the model, the transformed priming effect (*rt_recoded*) was predicted by the raw priming effect (*priming_rt*), SOA(*soa*), *session*, and a random intercept for each *participant*. The precision parameter (phi) was held constant across observations.

$$\text{rt\_recoded} \sim \text{priming\_rt} + \text{soa} + \text{session} + (1 \mid \text{participant})$$

$$\phi \sim 1$$

For the intercept and the fixed-effect regression coefficients of *priming_rt*, *soa*, and *session*, we used independent normal distributions centred on 0 with a standard deviation of 5 (Normal$(0{,}5)$), which are weakly informative. For the prior of the participant-specific random intercepts, we used a Student's (t) distribution with 3 degrees of freedom, a location of 0, and a scale of 2.5 (Student-t$(3{,}0{,}2.5)$), constrained to be non-negative. The model was fitted using the `brms` package in R with 4 chains of 3000 iterations each (1000 warmup). Bayes Factors were derived by testing whether the coefficient of *priming_rt* was zero as described in *Methods - Bayes Factors*.

## References

DeCarlo, L. T. (1998). Signal detection theory and generalized linear models. *Psychological Methods*, *3*(2), 186–205. https://doi.org/10.1037/1082-989X.3.2.186
